# Trisoxazole Macrolides Potentiate the Microtubule Assembly and Antimitotic Activities of Taxanes

**DOI:** 10.1002/anie.202522954

**Published:** 2026-03-17

**Authors:** Shohei Ebihara, Rio Takaiso, Shota Kawaguchi, Atsunori Oshima, Masaki Kita

**Affiliations:** ^1^ Graduate School of Bioagricultural Sciences Nagoya University Furo‐Cho Chikusa Nagoya Japan; ^2^ Graduate School of Pharmaceutical Sciences Nagoya University Furo‐Cho Chikusa Nagoya Japan; ^3^ Cellular and Structural Physiology Institute (CeSPI) Nagoya University Furo‐Cho Chikusa Nagoya Japan; ^4^ Institute For Glyco‐core Research (iGCORE) Nagoya University Furo‐Cho Chikusa Nagoya Japan; ^5^ Center For One Medicine Innovative Translational Research (COMIT) Nagoya University Furo‐Cho Chikusa Nagoya Japan; ^6^ Institute of Quantum Chemistry Innovation Institutes of Innovation for Future Society Nagoya University Furo‐Cho Chikusa Nagoya Japan; ^7^ Promotion Office for Open Innovation, Institutes of Innovation for Future Society Nagoya University Furo‐Cho Chikusa Nagoya Japan

**Keywords:** Marine trisoxazole macrolides, Microtubule assembly potentiation, Molecular modeling, Protein–protein interaction stabilizers, Synergistic antimitotic activity

## Abstract

Mycalolides are marine‐derived trisoxazole macrolides that exhibit potent actin‐depolymerizing activity and inhibit the proliferation, migration, and invasion of various cancer cells. The C25–C35 side‐chain moiety of mycalolides is important for their actin‐binding properties; however, the biological function of the C1–C24 trisoxazole macrolactone moiety remains largely unexplored. In this study, using a photoaffinity biotin probe, we identified tubulin as a new, actin‐independent target of mycalolide C (MyC). Ultracentrifugation, fluorescence‐based tubulin polymerization assays, and transmission electron microscopy of negatively stained microtubules (MTs) demonstrated that MyC and trisoxazole macrolactones exert potent synergistic effects on paclitaxel‐induced MT assembly and stabilization. These compounds enhanced the antiproliferative activity of paclitaxel by 4.2‐ to 6.7‐fold in HCT‐116 human colon cancer cells but showed little synergy in 3T3‐L1 murine fibroblast. Molecular modeling studies suggest that MyC stabilizes the protein–protein interactions between α/β‐tubulin heterodimers. Given that the C1–C24 trisoxazole macrolactone moiety and its analogues exhibit little cytotoxicity, our findings may contribute to the development of new lead compounds for MT inhibitors with reduced side effects.

## Graphical abstract

Photoaffinity experiments identified tubulin as a new target of mycalolides. Though weakly cytotoxic, the trisoxazole macrolactone moiety enhanced paclitaxel's microtubule assembly and antimitotic effects. Molecular modeling studies suggested that MyC stabilizes α/β‐tubulin heterodimer interactions. These findings may contribute to the development of novel microtubule inhibitors with fewer side effects.

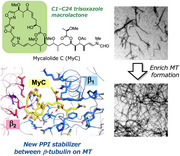

## 1 Introduction

Macrocyclic natural products are widely found in peptides, polyketides, terpenes, glycolipids, alkaloids, etc., and are derived from various organisms, including bacteria, archaea, and eukaryotes [1]. These compounds can specifically interact with target molecules and exhibit remarkable activities due to their specific conformation, rigidity, appropriate cell permeability, and stability. This provides useful information for the design and development of drugs, such as in the fields of cancer and infectious diseases [2].

**FIGURE 1:**
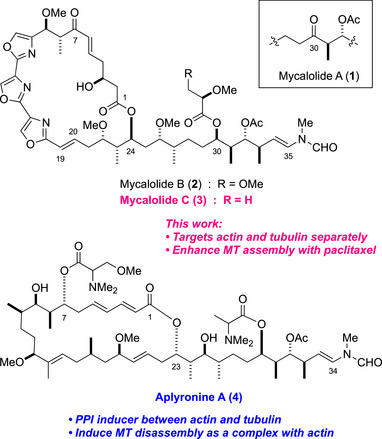
Structures of mycalolides A–C and aplyronine A.

Mycalolides A, B, and C (MyA–C, **1–3**) are cytotoxic and antimycotic trisoxazole macrolides isolated from the marine sponge *Mycale* sp. (Figure 1) [3, 4, 5, 6, 7, 8]. They inhibit actomyosin Mg^2+^‐ATPase [9] and show potent actin‐depolymerizing activity [10, 11]. Several trisoxazole macrolides that are structurally related to **1–3**, such as mycalolides D and E [12], ulapualides [13], halichondramides [14, 15, 16], jaspisamides [17], and kabiramides [18, 19] (Figure S1), similarly target actin and exhibit potent cytotoxicity, and some induce apoptosis in tumor cells [20, 21, 22]. MyB (**2**) also suppresses the proliferation, migration, and invasion of breast and ovarian cancer cells [23]. Thus, these unique macrocyclic compounds have been useful pharmacological tools for analyzing actin‐mediated cellular functions, such as muscle contraction, cell motility, and cell division.

Structure–activity relationship studies [24, 25, 26, 27, 28] and photolabeling experiments [29, 30] have revealed that the C25–C35 side‐chain moieties of mycalolides are important for their actin‐binding properties. In addition, x‐ray analyses of actin complexes with kabiramide C [31], jaspisamide A [31], ulapualide A [32], and more recently MyB [33] have shown that these side‐chain moieties commonly insert into actin to form a 1:1 complex. Mycalolides and their congeners have received considerable attention in the synthesis community, and total syntheses of MyA (**1**) [34, 35] and ulapualide A [36, 37, 38, 39, 40] have been reported [41]. We also achieved the synthesis of MyB (**2**) along with **1** using olefin metathesis as a key step [42]. In addition, the 19*E*‐ and 19*Z*‐macrolactone analogues of mycalolides have been synthesized, but they exhibited little cytotoxicity against tumor cells, scant actin‐depolymerizing properties, and low antimycotic activity against pathogenic fungi [43]. Thus, the biological functions of the C1–C24 macrolactone moiety of mycalolides and related trisoxazole compounds are still largely unknown.

Meanwhile, the mode of action of aplyronine A (ApA, **4**), an antitumor macrolide structurally related to mycalolides, has been studied [44, 45, 46, 47]. As with similar microfilament‐destabilizing macrolides (e.g., reidispongiolides and sphinxolides) (Figure S1) [48, 49], the actin‐depolymerizing activity of ApA was thought to be mostly important. However, studies using photoaffinity and fluorescent derivatives [20, 30, 50] have shown that ApA binds synergistically to tubulin in association with actin and inhibits spindle formation and mitosis in cancer cells [51, 52, 53, 54]. Interactions between microtubule (MT) and actin underlie many fundamental cellular processes, including cell motility, neuronal pathfinding, cell division, and cortical flow [55]. Although various proteins mediate MT–actin interactions and regulate their dynamics, ApA is noteworthy for being the first small molecule to stabilize actin and tubulin interactions by forming a 1:1:1 heterotrimeric complex [56, 57].

To precisely analyze target protein–ligand interactions, we have developed several tools, such as a fluorescent affinity MS tag *N*,*N*‐dimethylaminopyrene [58, 59] and photoreactive diazirine–alkyne tags [60]. Using them, we have searched for a target molecule other than actin that is important for the potent activity of mycalolides, and consequently identified tubulin and discovered their unique protein–protein interaction (PPI) stabilizing effects. Here we report that MyC (**3**) and its macrolactone analogs promote the MT assembly and antimitotic activities of paclitaxel in cancer cells.

## 2 Results and Discussion

### 2.1 Identification of Tubulin as the Second Target of MyC

**FIGURE 2:**
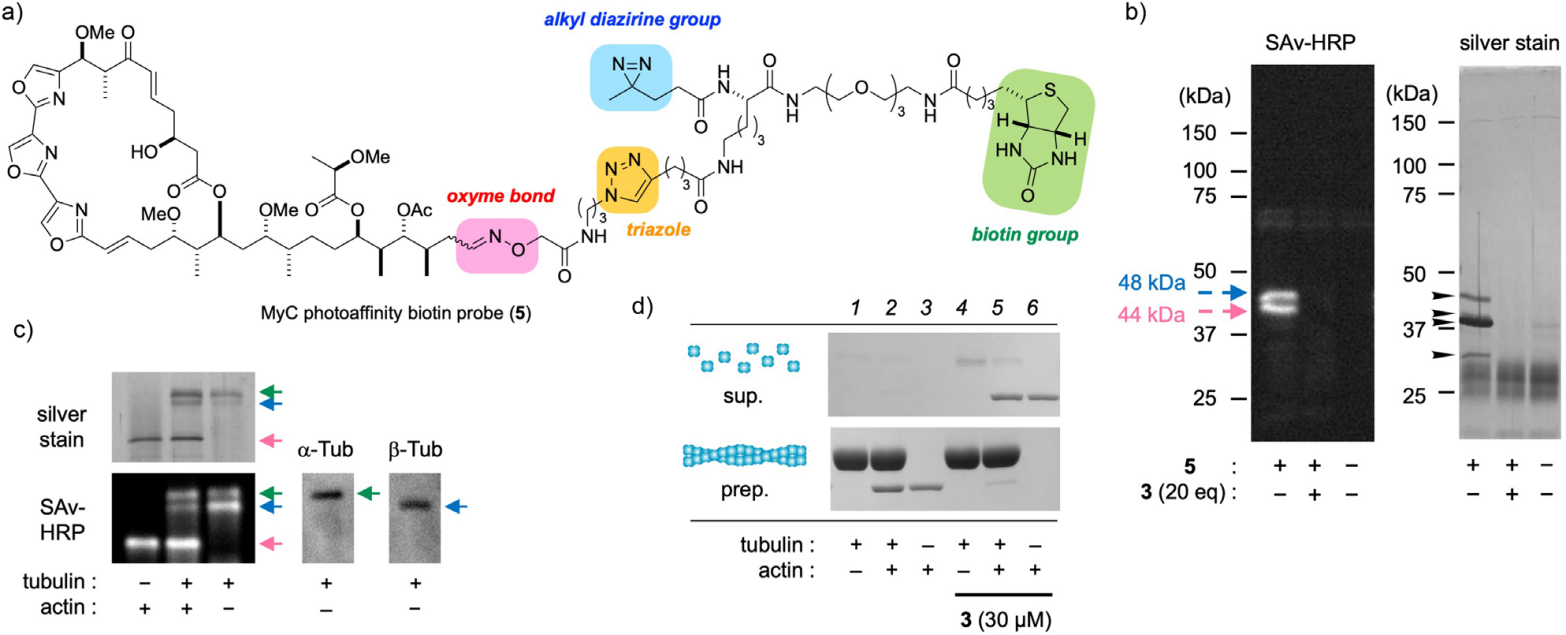
Target identification of MyC (**3**) by photoaffinity labeling and in vitro filamentous (F‐) actin and microtubule (MT) sedimentation assay. (a) Structure of the MyC photoaffinity‐biotin probe **5**. (b) Photolabeling and pull‐down experiments using the lysate of human colon cancer HCT116 cells. Labeled proteins were assigned by MS/MS analysis of their triptic peptides as β‐tubulin (48 kDa, blue) and actin (44 kDa, red), respectively. (c) Photolabeling experiments using purified actin (from rabbit muscle) and/or tubulin (from porcine brain). Labeled α‐ and β‐tubulin were assigned by immunoblotting analysis (green/blue arrows, respectively). (d) In vitro F‐actin and MT sedimentation assay. F‐actin and MT (3 µM as a α/β‐tubulin heterodimer) were precipitated by ultracentrifugation after treatment with or without **3** (30 µM). Proteins in the supernatant (sup.) and precipitate (prep.) were analyzed by SDS‐PAGE and detected with CBB stain.

According to previous derivatization and structure‐activity relationship studies on aplyronines and mycalolides [30, 61, 62] we designed the MyC photoaffinity biotin probe **5** (Figure 2a). Acidic hydrolysis of the *N*‐methyl enamide in MyC (**3**) and condensation with an alkoxyamine linker gave an azide derivative. Subsequent copper(I)‐catalyzed cycloaddition with an alkyne tag [60] provided **5** in 16% yield (3 steps, see the Supporting Information for details). After the photoreaction (365 nm) with probe **5** in the lysate of HCT116 human colon cancer cells, affinity purification using NeutrAvidin agarose and silver staining detected several target proteins (48, 44, and 30 kDa) as well as a major protein actin (42 kDa) (Figures 2b and S2). Blotting analysis with streptavidin‐horse radish peroxidase (SAv‐HRP) conjugate revealed two biotinylated proteins at 48 and 44 kDa, consistent with the silver staining results. Mass analysis of tryptic peptide fragments established that the 48 and 44 kDa bands contained β‐tubulin and actin, respectively, whereas the 30 kDa band consisted mainly photodegraded actin (see the Supporting Information for details). Furthermore, competition experiments with excess MyC (**3**) revealed that all photolabeled and/or affinity‐purified protein bands almost disappeared (Figures 2b and S2), suggesting that the bindings of probe **5** to these proteins were highly specific.

Next, photoaffinity labeling between MyC (**3**), actin, and tubulin was examined using purified proteins. Probe **5** formed a covalent bond with porcine brain tubulin regardless of the presence of rabbit muscle actin (Figures 2c and S2). Unlike the experiments with human cell lysates, tubulin was detected as two separate bands by both SAv‐HRP detection and silver staining. Immunoblot analysis revealed that these protein bands corresponded to α‐ and β‐tubulins, respectively. These results suggest that MyC binds independently to tubulin and actin and has a different mechanism of action from ApA (**4**), which binds to α/β‐tubulin heterodimer only in the presence of actin [51].

### 2.2 MT Assembly and Stabilizing Effect of MyC and Trisoxazole Compounds

First, filamentous (F‐) actin and MT sedimentation activity of MyC (**3**) was examined using an ultracentrifugation method [30, 63]. As expected from its potent actin‐depolymerizing activity, treatment with excess MyC reduced the amount of F‐actin in the precipitate (prep.) fractions, and the protein bands were mostly (>90%) observed in the supernatant (sup.) fractions (*lanes 2, 3 vs lanes 5, 6*) (Figures 2d and S3). Previous studies have revealed that ApA specifically induces MT disassembly in the presence of actin, and both proteins were dominantly detected in the sup. fraction [64]. In contrast, MyC did not cause MT disassembly even at sufficiently high concentrations, and most of the tubulin (>95%) remained in the prep. fractions regardless of the presence of actin (*lanes 1, 2 vs lanes 4, 5*). These results suggest that MyC does not depolymerize MTs, unlike ApA.

**FIGURE 3:**
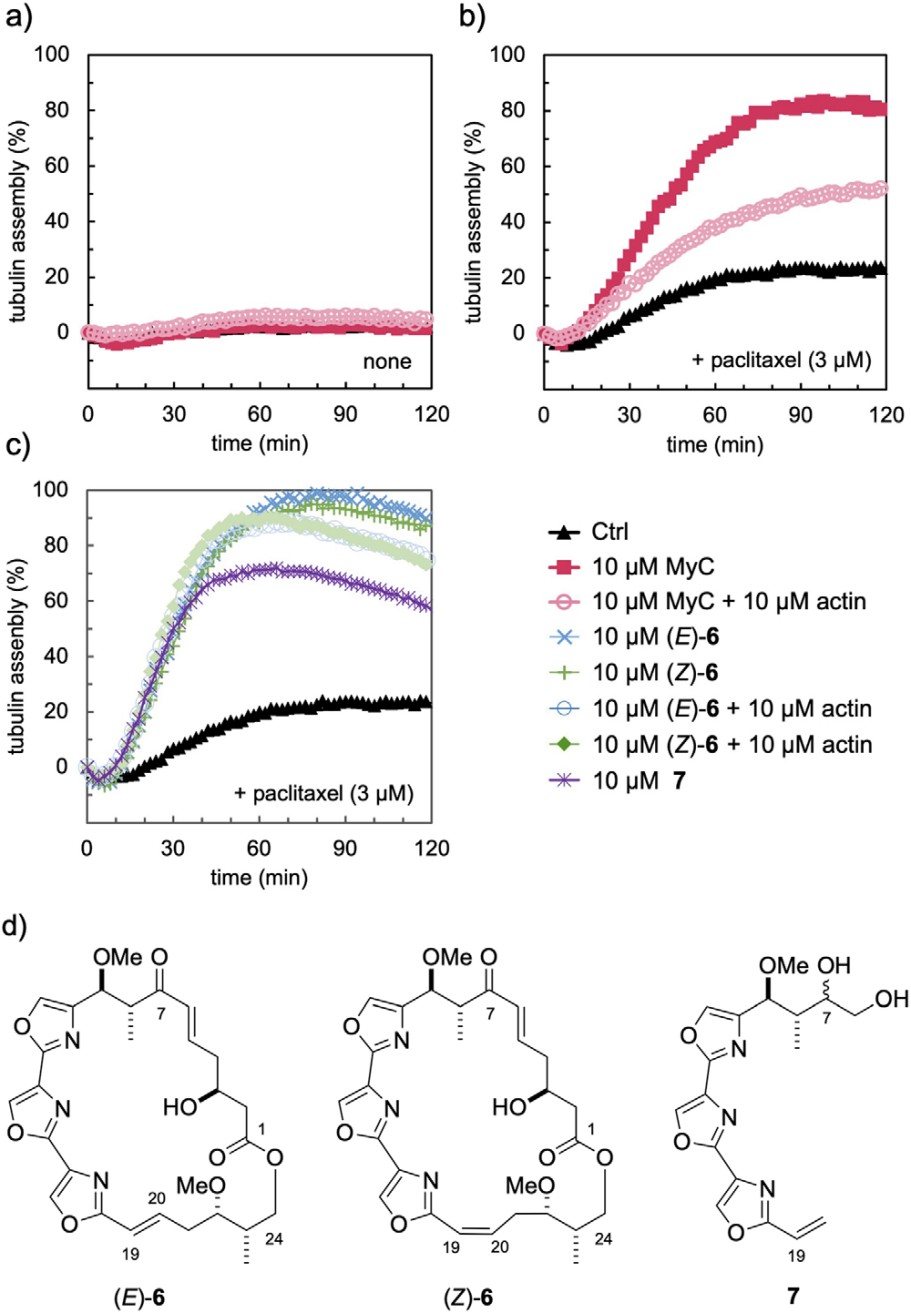
In vitro tubulin polymerization assay using α/β‐tubulin heterodimer. Tubulin (18 µM for the heterodimer) was polymerized in assay buffer [80 mM PIPES·Na (pH 6.9), 2 mM MgCl_2_, 0.5 mM EGTA, 1 mM GTP, 4.6 µM DAPI] at 37°C, as monitored by an increase in DAPI fluorescence (λ_ex_/λ_em_ 360/450 nm). (a) MyC (**3**) and/or actin treatment without paclitaxel. (b) MyC (**3**) and/or actin treatment with 3 µM paclitaxel. (c) Trisoxazole analog treatment with 3 µM paclitaxel. (d) Structures of trisoxazole analogs **6** and **7**.

To investigate the effect of MyC on MT assembly, we next examined a fluorescence‐based tubulin polymerization assay, in which 20% glycerol induces rapid polymerization (maximal assembly reached within 30 min) [65]. Without glycerol, treatment of 10 µM MyC with tubulin (18 µM) did not cause any MT assembly (Figure 3a), while 3 µM paclitaxel induced a slow and partial polymerization, with the relative assembly rate reaching approximately 20% at 2 h (Figure 3b). Notably, the combination of 10 µM MyC and 3 µM paclitaxel significantly promoted MT assembly (ca. 80% in 90 min), whereas the further addition of 10 µM actin rather inhibited assembly (ca. 50% in 90 min). These results suggest that MyC exerts a potent synergistic effect on paclitaxel‐induced MT assembly and stabilization.

The *K*_D_ value for the actin–MyB complex is 13–20 nM, [10] and a similar *K*_D_ is expected for the actin–MyC complex. In tubulin polymerization assays (Figure 3b), the MyC enhancing effect was only partially inhibited by actin even at a 1:1 molar ratio. Moreover, in the photolabeling experiments using probe **5** (Figure 2c), the relative labeling yields of α/β‐tubulin and actin were nearly identical (0.97:1.00) by densitometric analysis (Figure S2c). These results suggest that the MyC binds to MT or tubulin heterodimers with an affinity comparable to, though slightly weaker than, its actin binding.

Because the C25–C35 side‐chain moiety is sufficient for the actin‐depolymerizing activity of mycalolides, [24, 25] we predicted that the C1–C24 macrolide moiety is responsible for MT assembly. Therefore, tubulin polymerization assays were examined using MyC macrolactone analog (*E*)‐**6**, its C19 geometric isomer (*Z*)‐**6** [43, 66] and the C7–C19 segment **7** (a synthetic intermediate of mycalolides) [67] (Figures 3c and 3d). Indeed, the combination of 10 µM (*E*)‐**6** or (*Z*)‐**6** with 3 µM paclitaxel also promoted MT assembly highly efficiently (90∼95% in 90 min), while the addition of 10 µM actin to these systems had a slight effect (80∼85% in 90 min). Moreover, 10 µM **7** promoted MT assembly to a similar extent as MyC (**3**) (ca. 70% in 60 min). These results suggest that the trisoxazole moiety of MyC may be essential for its synergistic effect with paclitaxel on MT formation and stabilization.

**FIGURE 4:**
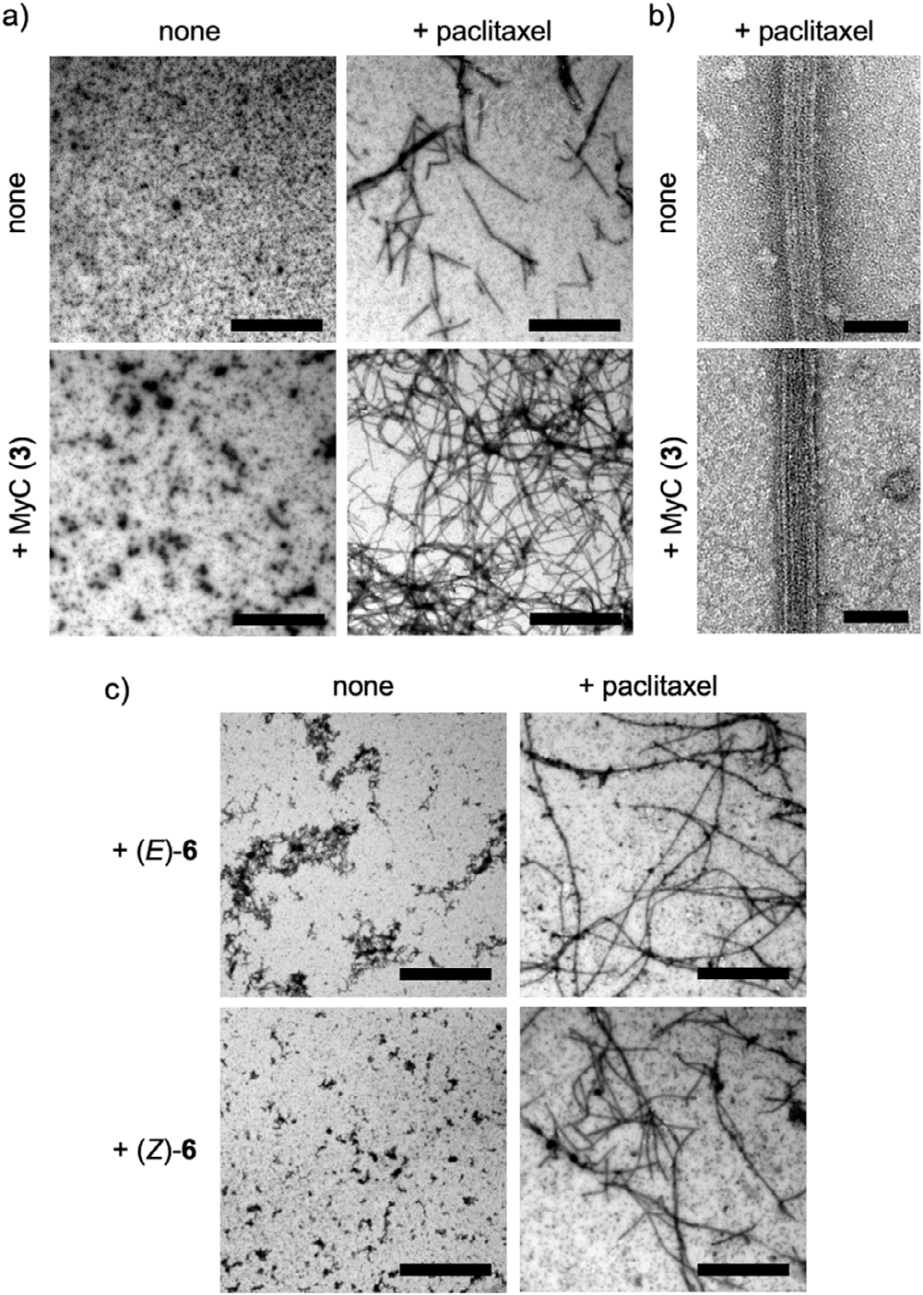
In vitro tubulin polymerization assay. MTs were prepared by the polymerization of tubulin (3 µM) with paclitaxel (6 µM) and/or trisoxazole compounds (8.5 µM) for 30 min at 37°C. The polymerized MTs were negatively stained and observed with TEM. (a), (c) Scale bar = 3 µm (× 5,000 zoom). (b) Scale bar = 50 nm (× 40,000 zoom).

To visualize the effects of MyC (**3**) and paclitaxel on MT assembly, we performed negative stain transmission electron microscope (TEM) analysis of the MTs prepared with tubulin (3 µM as α/β‐heterodimer). In the absence of paclitaxel, treatment with MyC (8.5 µM) led to the formation of oligomeric tubulin aggregates, but no filamentous structures were observed (Figure 4a). In the presence of paclitaxel (6 µM), MTs with rod‐like structures were formed, although their lengths were limited. On the other hand, simultaneous treatment with MyC and paclitaxel significantly promoted the elongation of MT filament structures. The average diameter of MTs was not significantly different in the absence or presence of MyC (24.90 ± 0.56 nm and 24.87 ± 0.55 nm, respectively) (Figure 4b). Furthermore, combined treatment with macrolactones (*E*)‐**6** or (*Z*)‐**6** (8.5 µM) and paclitaxel also enhanced the length of MT filamentous structures (Figure 4c), consistent with the results of the fluorescence‐based tubulin polymerization assay as described above.

### 2.3 Synergistic Antiproliferative Effect of Trisoxazole Macrolactones and Paclitaxel on Cancer Cells

Paclitaxel and MyC (**3**) alone showed antiproliferative activity against HCT‐116 cells (IC_50_ = 3.1 and 20 nM, respectively), whereas the addition of 100 pM of **3** enhanced the activity of paclitaxel 4.2‐fold (Table 1). Similarly, the macrolactones (*E*)‐**6** and (*Z*)‐**6** alone showed little growth inhibitory effect (IC_50_> 1 µM), but their addition at 100 nM enhanced the activity of paclitaxel 6.7‐ and 5.0‐fold, respectively. Furthermore, even at concentrations lower than the IC_50_ values, both (*E*)‐**6** and (*Z*)‐**6** significantly enhanced the activity of 0.1 and 0.3 nM paclitaxel (Figure S4). These clearly demonstrated that trisoxazole macrolides and paclitaxel, which synergistically enhance MT assembly, inhibit cancer cell growth based on their antimitotic effects.

**TABLE 1:** Synergistic growth inhibitory effects of trisoxazole macrolactones and paclitaxel on human colon cancer HCT116 cells. The difference in sensitivity compared to murine fibroblast 3T3‐L1 cells is shown as a ratio.

Compound	Additive	IC_50_ (nM)		Ratio (fold)
		HCT116	3T3‐L1	
Paclitaxel	—	3.1	204	66
MyC (**3**)	—	20	178	8.9
(*E*)‐**6**	—	>1000	>1000	—
(*Z*)‐**6**	—	>1000	>1000	—
Paclitaxel	100 pM **3**	0.74	—	260
	1 nM **3**	—	196	
	100 nM (*E*)‐**6**	0.46	264	570
	100 nM (*Z*)‐**6**	0.62	280	450

We also examined the antiproliferative activity against the murine fibroblast 3T3‐L1 as a noncancer cell line. Paclitaxel and MyC alone were less potent than in HCT‐116 cells (IC_50_ = 204 and 178 nM, respectively), and little synergy was observed upon co‐treatment with **3** or **6** (Table 1). Consequently, the cancer‐to‐normal cell activity ratios increased from 66‐fold (paclitaxel alone) to 260‐, 570‐, and 450‐fold with **3**, (*E*)‐**6**, and (*Z*)‐**6**, respectively. Since HCT116 cells proliferate rapidly and are more sensitive to MT inhibitors than normal cells, it is reasonable to speculate that these trisoxazole compounds, together with paclitaxel, synergistically inhibited MT dynamics in the cells.

We further evaluated A549 (lung), MCF‐7 (breast), and L1210 (leukemia) cancer cells, but no synergy was observed in these cell lines (Figures S4 and S5; Table S1). Although the mechanism underlying this HCT116‐specific synergy remains unclear, we plan to investigate it further, including analyses of compound uptake and intracellular accumulation.

Previous live‐cell imaging studies showed that fluorescently labeled MyB (**2**) and ApA (**4**) rapidly accumulate in the cytoplasm and remain intracellularly even after removal of the probes [20]. It is therefore plausible that MyC also exhibits highly cell permeability and cytoplasmic retention via actin binding, thereby enhancing paclitaxel activity even at 0.1 nM. In contrast, (*E*/*Z*)‐**6** lack actin‐binding tail and require higher concentration (100 nM) to achieve synergy, suggesting lower intracellular accumulation. Thus, both MyC and **6** may synergistically promote MT polymerization with paclitaxel in cancer cells.

### 2.4 Molecular Modeling Studies

**FIGURE 5:**
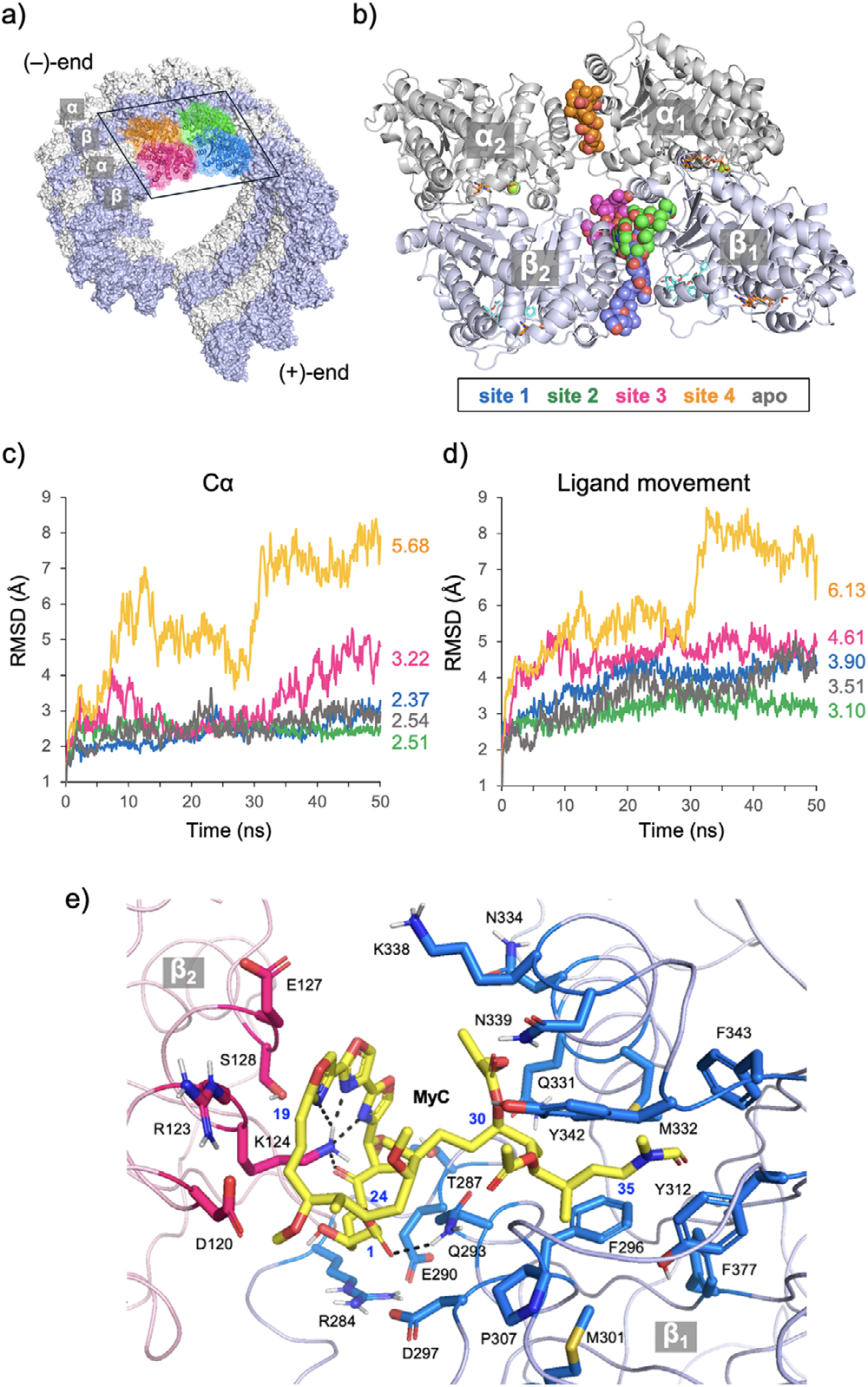
Proposed binding modes of MyC (**3**) on MT by docking and MD simulations. (a) Positions of the two pairs of α/β‐tubulin heterodimers on porcine MT (PDB: 5SYF). (b) Superposition of the lowest‐energy conformers of MyC at sites 1∼4 on tubulin heterotetramer. (c,d) MD simulations of the tubulin heterotetramer–MyC complex models. Conformational stability (c) and ligand movement dynamics (d) were simulated at 37°C, pH 7.4 for 50 ns. (e) Detailed interactions of MyC with tubulins (at site 2) proposed by docking simulation. The residues of β_1_‐ and β_2_‐tubulin interact with MyC (yellow) are shown in marine and hot pink stick models.

Tubulin polymerization is directional, with β‐tubulin facing the polymerization‐promoting plus‐end, and MTs are formed by the assembly of 13 α/β‐tubulin heterodimers (protofilaments), which then associate laterally to form a cylindrical structure (Figure 5a) [68]. Based on the hypothesis that MyC serves as a molecular glue to stabilize PPI between α/β‐tubulin heterodimers, we performed docking simulations using two pairs of α/β‐tubulin heterodimers (tubulin heterotetramers) arranged side‐by‐side in the cryo‐EM structure of MT stabilized with paclitaxel (PDB: 5SYF) [69]. Among the top 10 binding sites obtained using the Molecular Operating Environment (MOE) 2024.06 program package (Figure S6a), six highly overlapped those of paclitaxel, GTP, and GDP, which originally bind to tubulin. Therefore, the remaining four were designated as ligand‐binding pockets (sites 1–4), and conformational searches were performed using the Amber14:EHT force field. In the obtained models, MyC was mainly localized at the interface of the two heterodimers, but the poses were highly diverse (Figure S6b), with the lowest‐energy conformers ranging from –12.64 to –10.85 kcal/mol (Figures 5b and S6c). The site 4 model surrounded by two α‐tubulins (α_1_/α_2_) showed the best S scores (lowest in energy), followed by the site 2 and 3 models surrounded by two β‐tubulins (β_1_/β_2_). The site 1 model bound to the outside (plus ends) of two β‐tubulins was the least stable.

To further assess the docking simulation results, additional combinations of tubulin heterotetramer models and ligands were examined. Peloruside A, a cytotoxic macrolide isolated from the marine sponge *Micale* sp [70], stabilizes tubulin assembly by binding to the outer surface of MTs at the interface between two β‐tubulin subunits and exhibits potent antitumor activity [71, 72]. As an MT inhibitor distinct from taxanes, peloruside A has also been explored as a potential lead compound for the treatment of neurodegenerative and autoimmune diseases [73, 74]. Docking simulations of MyC with tubulin heterotetramers containing peloruside A, in the absence or presence of paclitaxel (PDB: 5SYC and 5SYE) [69], revealed that the site 1–3 and site 1–2 models were more stable than the site 4 model (Figure S7). In contrast, docking simulations of peloruside A with the original tubulin heterotetramer (PDB: 5SYF) favored the site 4 model rather than the experimentally validated site 2 model by cryo‐EM analysis (Figure S8). These results indicate that docking scores alone are insufficient to reliably predict the correct binding modes.

We therefore performed molecular dynamics (MD) simulations of the optimal site 1–4 models using YASARA software under conditions mimicking the cellular environment (density 0.997 g/mL, 310 K, and pH 7.4). After 50 ns of simulation, the site 2 model showed the highest stability in terms of both conformational stability (C_α_, average RMSD 2.51 Å) (Figure 5c) and ligand movement dynamics (average RMSD 3.10 Å) (Figure 5d). In contrast, the site 3 and 4 models showed considerably larger fluctuations in both terms. The average total number of PPI residues between α_1_/α_2_ and β_1_/β_2_ tubulins in the site 2 model remained almost unchanged throughout the 50 ns MD simulation, similar to those of the apo and site 1 models (Figure S9). In contrast, in the site 3 and 4 models, PPIs on the opposite side of the MyC‐binding interface were greatly reduced, which may destabilize these complexes due to the increased distance between the two heterodimers.

For comparison, MD simulation was also performed for the tubulin heterotetramer model with peloruside A (PDB: 5SYC). The average RMSD values for both conformational stability and ligand movement dynamics were small (2.38 and 2.08 Å, respectively), and the number of PPI residues between α_1_/α_2_ and β_1_/β_2_ tubulins was well maintained (Figure S10). These results suggest that the site 2 model represents the most plausible binding mode for MyC. In this model, the macrolactone moiety is surrounded by helix 2 (D120–S128) of β_2_ and helices 9 (T287–D297) and 10 (Q331–N339) of β_1_, in which the K124 amino group forms electrostatic interactions with three oxazole nitrogen atoms and the C7 ketone, while the Q293 amide group interacts with the C1 ester group (Figure 5e). In addition, the side‐chain moiety of MyC adopts a bent conformation and is stably accommodated mainly through hydrophobic interactions with residues in the coil structure of β_1_, including Q293, F296, Q331, M332, N339, Y342, F377. Notably, the interacting residues with the macrolactone and side‐chain moieties of MyC were highly similar to those in peloruside A binding (Figure S11). Therefore, stabilization of tubulin heterodimer interactions by MyC might enhance the MT‐stabilizing effect of paclitaxel and synergistically inhibit mitosis in HCT116 cells, even at concentrations lower than intracellular tubulin levels.

## 3 Conclusion

In summary, we identified tubulin as a new target molecule for mycalolides, which have been thought to be cytotoxins that only target actin. We showed that MyC (**3**) and trisoxazole compounds **6** and **7** promote and stabilize paclitaxel‐induced MT assembly and that compounds **3** and **6** markedly exhibit synergistic inhibitory effects on the proliferation of HCT‐116 cancer cells, demonstrating 20–25% inhibition even below the IC_50_ values (Figure S4).

Tubulin is one of the most abundant cytoplasmic proteins, with an intracellular concentration of approximately 20 µM [56]. The concentrations of **3** ( 0.1 nM) and **6** (100 nM) that produced synergistic effects were therefore far below the endogenous tubulin level. Paclitaxel inhibits mitosis by stabilizing the dynamics of mitotic spindles without altering the total mass of polymerized MTs, even at concentrations lower than that of tubulin [75, 76]. These observations suggest that the trisoxazole compounds may synergistically potentiate paclitaxel activity at low concentrations by interacting with MT plus ends.

Our findings suggest that various previously discovered trisoxazole macrolides, such as kabiramides, ulapualides, and halichondramides (Figure S1), may also target tubulin in a similar manner to mycalolide and synergistically enhance the activity of paclitaxel. Taxane drugs such as paclitaxel, docetaxel, and cabazitaxel have been widely used in cancer chemotherapy, but since they target MTs, side effects such as neurological disorders and acute pain syndrome are a serious problem [77, 78]. Peloruside A and taxanes have been shown to functionally synergize in inhibiting cancer cell proliferation [79, 80]. Nevertheless, our finding that low concentrations of MyC and trisoxazole compounds enhance the activity of paclitaxel is very promising. Because trisoxazole compounds show little cytotoxicity, if they can be localized in cancer tissues by prior administration, it may be possible to increase the local sensitivity to taxane drugs and reduce their side effects. In addition, hybrid molecules artificially linking trisoxazoles and taxanes are attracting attention as lead compounds for unprecedented MT inhibitors to overcome taxane resistance in cancer [81, 82, 83]

Combination of the docking and MD simulations proposed that MyC binds between the two β‐tubulins on MT, which corresponds to the result that β‐tubulin was selectively photolabeled and affinity‐purified in human cell lysate. However, despite the identical sequences of the major α‐ and β‐tubulins between humans and pigs, photolabeling and pull‐down experiments using human cell lysates and porcine brain tubulin gave slightly different results. It is conceivable that endogenous associated proteins that bind to MTs, such as motor proteins, contained in the lysate may influence the selectivity and stability of tubulins. In the future, we hope to more precisely analyze the binding mode of the MT–MyC complex using cryo‐electron microscopy and ligand‐dissociation‐type probes [58, 59] to establish the possible synergistic effect of MyC in living cells, and to develop new ligands that have simpler structures yet are more effective at stabilizing PPIs. The challenge of promoting chemical biology research that elucidates the unknown functions of marine macrocyclic compounds beyond the relationships between organisms and creating breakthroughs that overturn conventional wisdom, as in this study, will contribute to the revival of drug lead development using natural products as new seeds.

## Conflicts of Interest

The authors declare no conflicts of interest.

## Supporting information




**Supporting File 1**: anie71854‐sup‐0001‐SuppMat.pdf.

## Data Availability

The data that support the findings of this study are available in the Supporting Information of this article.
