# Supplementary material for: Trisoxazole Macrolides Potentiate the Microtubule Assembly and Antimitotic Activities of Taxanes
Source: Angew Chem Int Ed Engl. 2026 Mar 17;65(18):e22954. doi: 10.1002/anie.202522954 (PMC13110761; doi:10.1002/anie.202522954)
Supplement: Supplementary file 1 — Supporting File 1: anie71854‐sup‐0001‐SuppMat.pdf. [file ANIE-65-e22954-s001.pdf]

Supporting Information

©Wiley-VCH 2025

69451 Weinheim, Germany

Trisoxazole Macrolides Potentiate the Microtubule Assembly and Antimitotic Activities of Taxanes

Shohei Ebihara, Rio Takaiso, Shota Kawaguchi, Atsunori Oshima, and Masaki Kita *

Abstract: Mycalolides are marine-derived trisoxazole macrolides that exhibit potent actin-depolymerizing activity and inhibit the proliferation, migration, and invasion of various cancer cells. The C25–C35 side-chain moiety of mycalolides is important for their actin-binding properties; however, the biological function of the C1–C24 trisoxazole macrolactone moiety remains largely unexplored. In this study, using a photoaffinity biotin probe, we identified tubulin as a new, actin-independent target of mycalolide C (MyC). Ultracentrifugation, fluorescence-based tubulin polymerization assays, and transmission electron microscopy of negatively stained microtubules (MTs) demonstrated that MyC and trisoxazole macrolactones exert potent synergistic effects on paclitaxel-induced MT assembly and stabilization. These compounds enhanced the antiproliferative activity of paclitaxel by 4.2- to 6.7-fold in HCT-116 human colon cancer cells but showed little synergy in 3T3-L1 murine fibroblast. Molecular modeling studies suggest that MyC stabilizes the protein–protein interactions between α/β-tubulin heterodimers. Given that the C1–C24 trisoxazole macrolactone moiety and its analogues exhibit little cytotoxicity, our findings may contribute to the development of new lead compounds for MT inhibitors with reduced side effects.

DOI: 10.1002/anie.2026XXXXX

Contents

Supporting figures and tables	S1~S12
Materials and methods	S13~S17
PMF analysis	S18~S21
NMR and Mass spectra	S22~S24
HPLC chart	S24
Original gel images	S25
Supporting references	S25

Supporting Figures

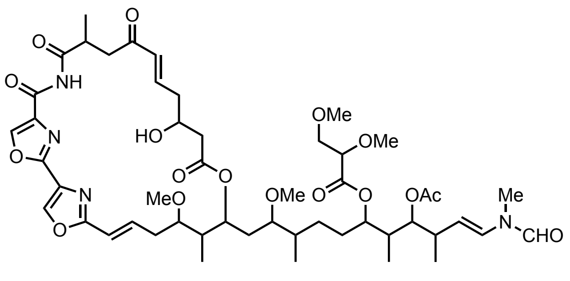

Mycalolide D

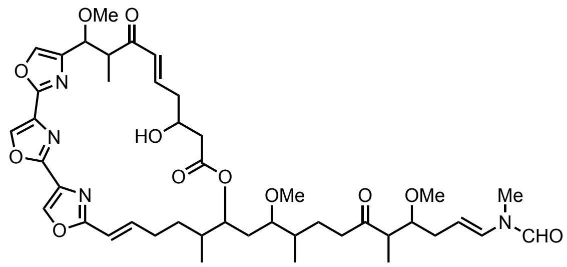

Halichondramide

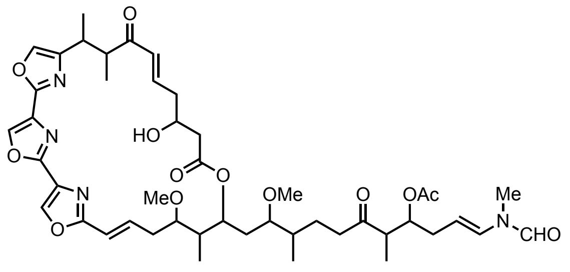

Mycalolide E

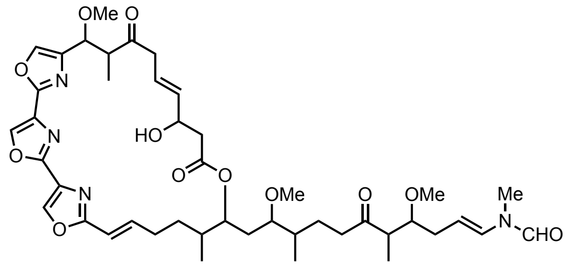

Jaspisamide A

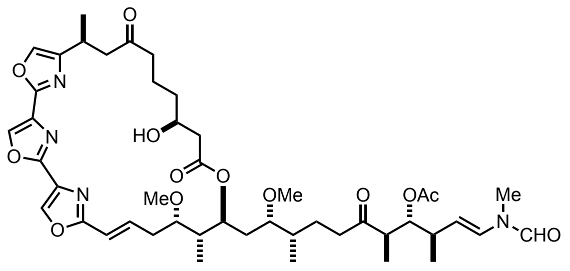

Ulapualide A

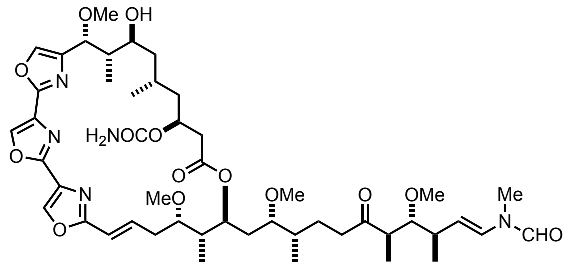

Kabiramide C

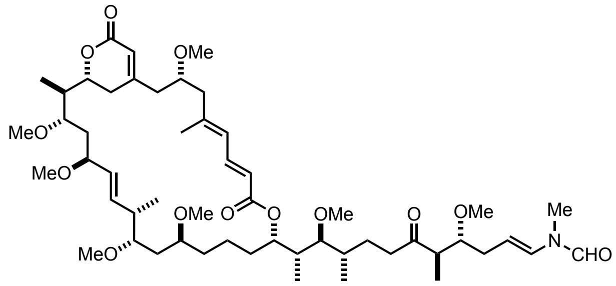

Reidispongiolide A

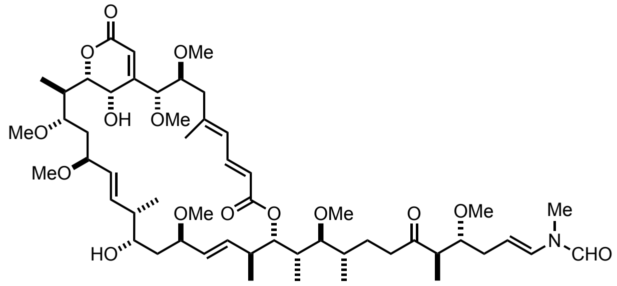

Sphinxolide A

Figure S1. Structures of representative microfilament-destabilizing macrolides related to mycalolides A–C.

A

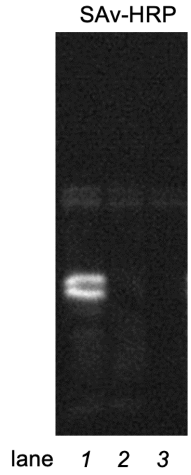

lane	1	2	3
48 kDa	0.90	0.03	0.01
44 kDa	1.00	0.02	0.01

Fig. 2B

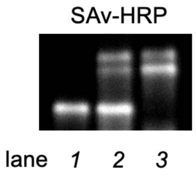

lane	1	2	3
α-tublin	–	0.51	0.48
β-tublin	–	0.46	0.81
actin	0.81	1.00	–

Fig. 2C

B

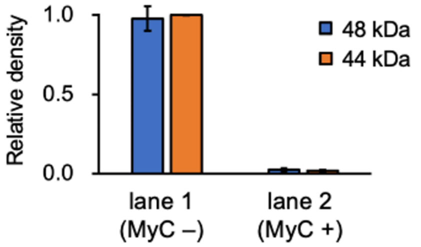

Fig. 2B

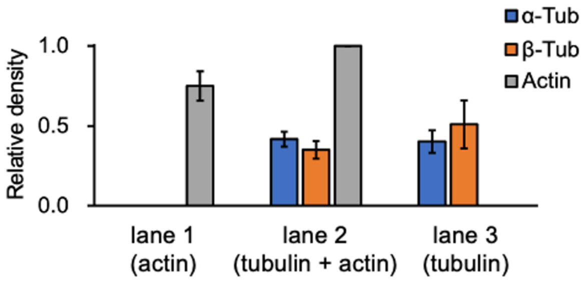

Fig. 2C

Figure S2. Quantitative blot analysis. a) Densitometric analysis of the blotting data in Figs. 2b and 2c. In each panel, relative density values are shown. b) Comparison of the density of photolabeled actin and tubulin bands for the experiments in Figs. 2b (lanes 1 and 2, n = 2) and 2c (lanes 1–3, n = 3). Values are shown as the mean ± SD.

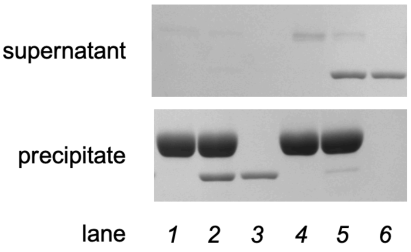

	lane	1	2	3	4	5	6
supernatant	tublin	0.017	0.020	–	0.049	0.010	–
	actin	–	0.013	0.011	–	0.146	0.149
precipitate	tublin	0.965	1.000	–	0.953	0.958	–
	actin	–	0.202	0.164	–	0.016	0.003
precipitate ratio (%)	tubulin	98.3	98.0	–	95.1	98.9	–
	actin	–	93.9	93.8	–	10.0	1.7

Figure S3. Densitometric analysis of the CBB stain in Fig. 2d. In each panel, relative density values are shown.

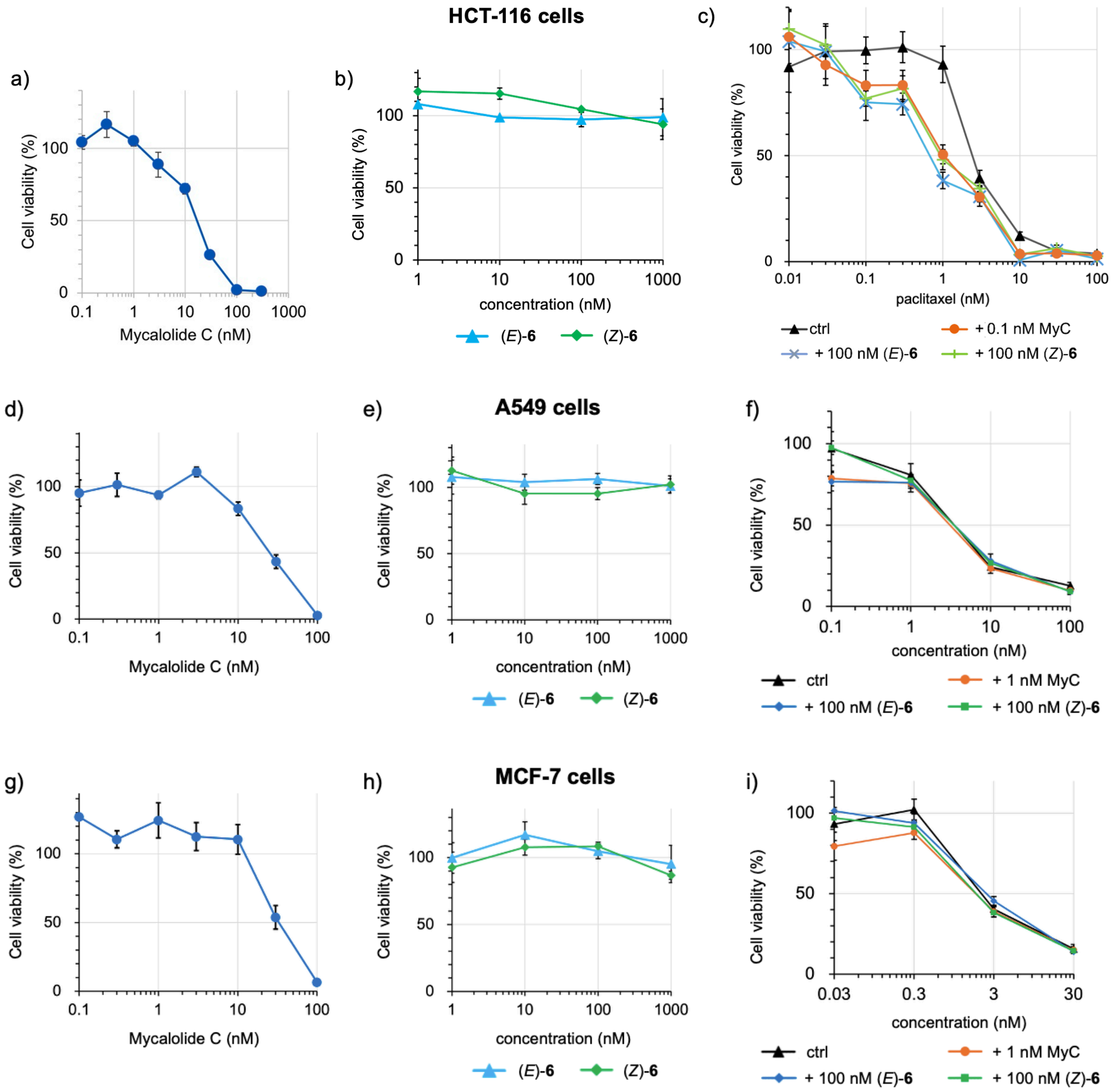

Figure S4. Synergistic effects of tris-oxazole macrolactones on the cytotoxicity of paclitaxel against human cancer cells. The data are expressed as the mean ± SD (n = 4). Statistical analyses were performed using Dunnett's multiple comparison tests. IC_{50} values are shown in Tables 1 and S1. a, b) Cytotoxicity of MyC, (*E*)-**6**, and (*Z*)-**6** alone against HCT116 colon cancer cells. c) Cytotoxicity of paclitaxel in the absence (shown as ctrl) and presence of additives against HCT116 cells. Both (*E*)-**6** and (*Z*)-**6** significantly enhanced the growth inhibitory activity of 0.1, 0.3, and 1 nM paclitaxel ($P < 0.05$). MyC also significantly enhanced this activity of 0.3 and 1 nM paclitaxel ($P < 0.05$). d–f) Cytotoxicity of each compound in the absence and presence of paclitaxel against A546 lung cancer cells. g–i) Cytotoxicity of each compound in the absence and presence of paclitaxel against MCF7 breast cancer cells.

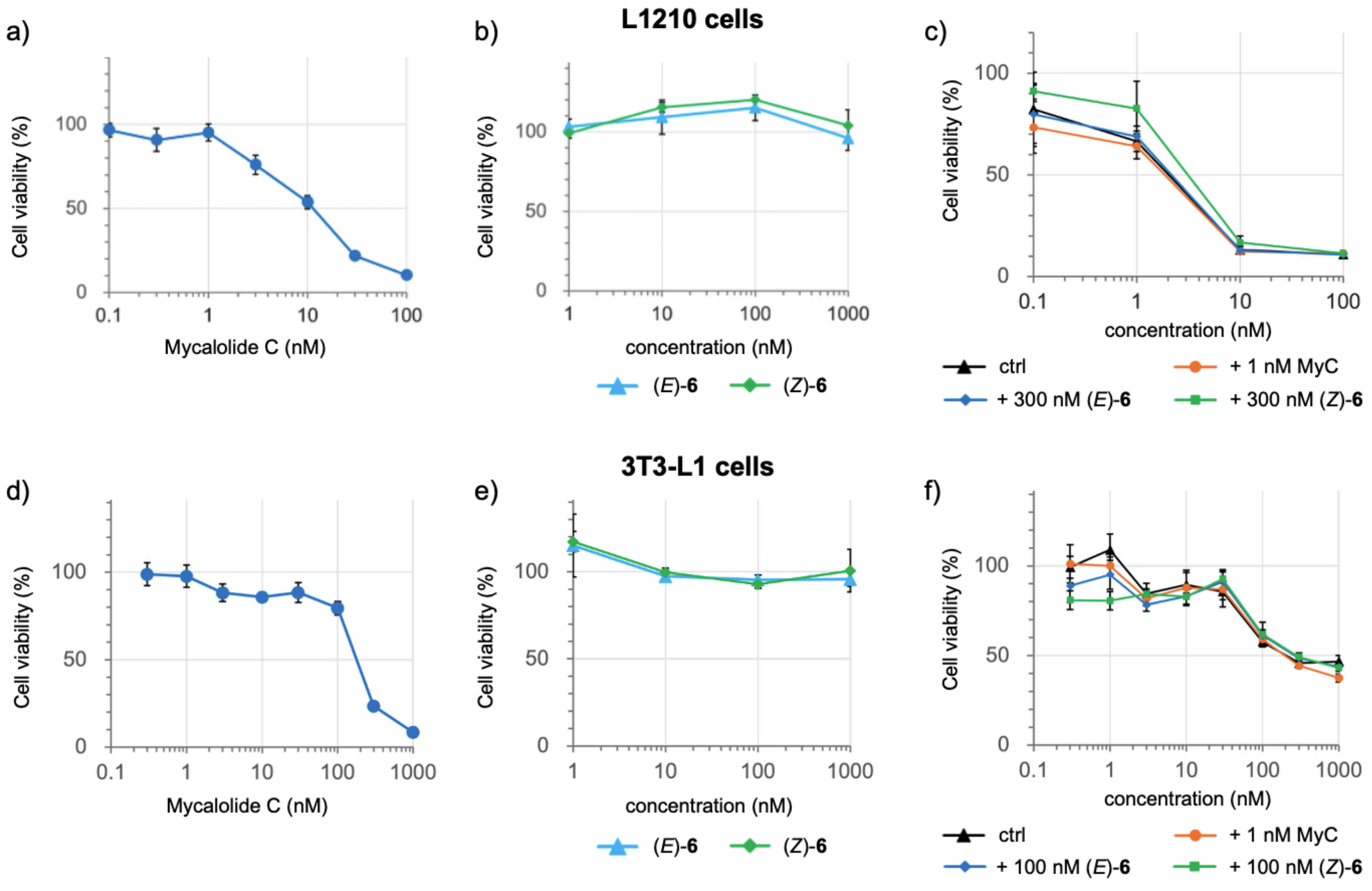

Figure S5. Synergistic effects of tris-oxazole macrolactones on the cytotoxicity of paclitaxel against murine cancer and non-cancer cells. The data are expressed as the mean ± SD (n = 4). Statistical analyses were performed using Dunnett's multiple comparison tests. IC_{50} values are shown in Tables 1 and S1. a,b) Cytotoxicity of MyC, (*E*)-**6**, and (*Z*)-**6** alone against murine leukemia L1210 cells. c) Cytotoxicity of paclitaxel in the absence (shown as ctrl) and presence of additives against L1210 cells. d–f) Cytotoxicity of each compound in the absence and presence of paclitaxel against murine fibroblast 3T3-L1 cells. In both cells, MyC, (*E*)-**6** or (*Z*)-**6** did not significantly enhance the growth inhibitory activity of paclitaxel.

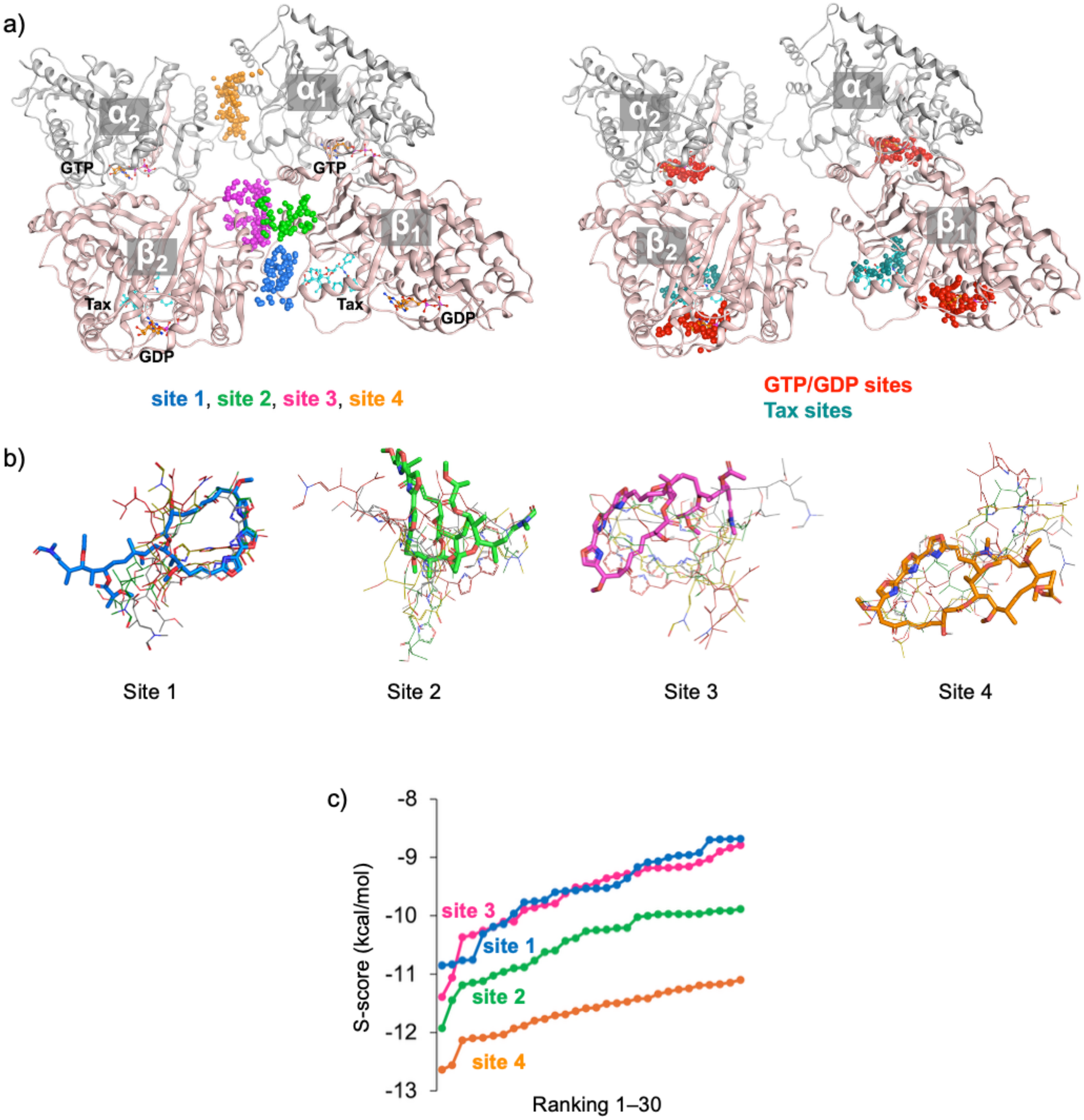

Figure S6. Docking simulation of MyC (**3**) with the tubulin heterotetramer prepared from the cryo-EM structure of MT with paclitaxel (PDB code: 5SYF). a) Overlayed structures of the top 10 binding sites on the tubulin heterotetramer. The four ligand binding sites 1 (blue), 2 (green), 3 (magenta), and 4 (orange) located between the two α/β-heterodimer pairs are highlighted in sphere models, along with the binding sites for paclitaxel (teal) and GTP/GDP (red). b) Superposition of the five lowest-energy conformers of MyC on the tubulin heterotetramer within 0.54, 0.81, 1.13, and 0.55 kcal/mol for sites 1–4, respectively. The most stable MyC conformer in each model is highlighted in stick models with the same colors as in a). c) Posing and scoring of the top 30 docking models of sites 1–4 based on the S-scores. Further MD simulations were performed for each complex with rank 1 (leftmost dots).

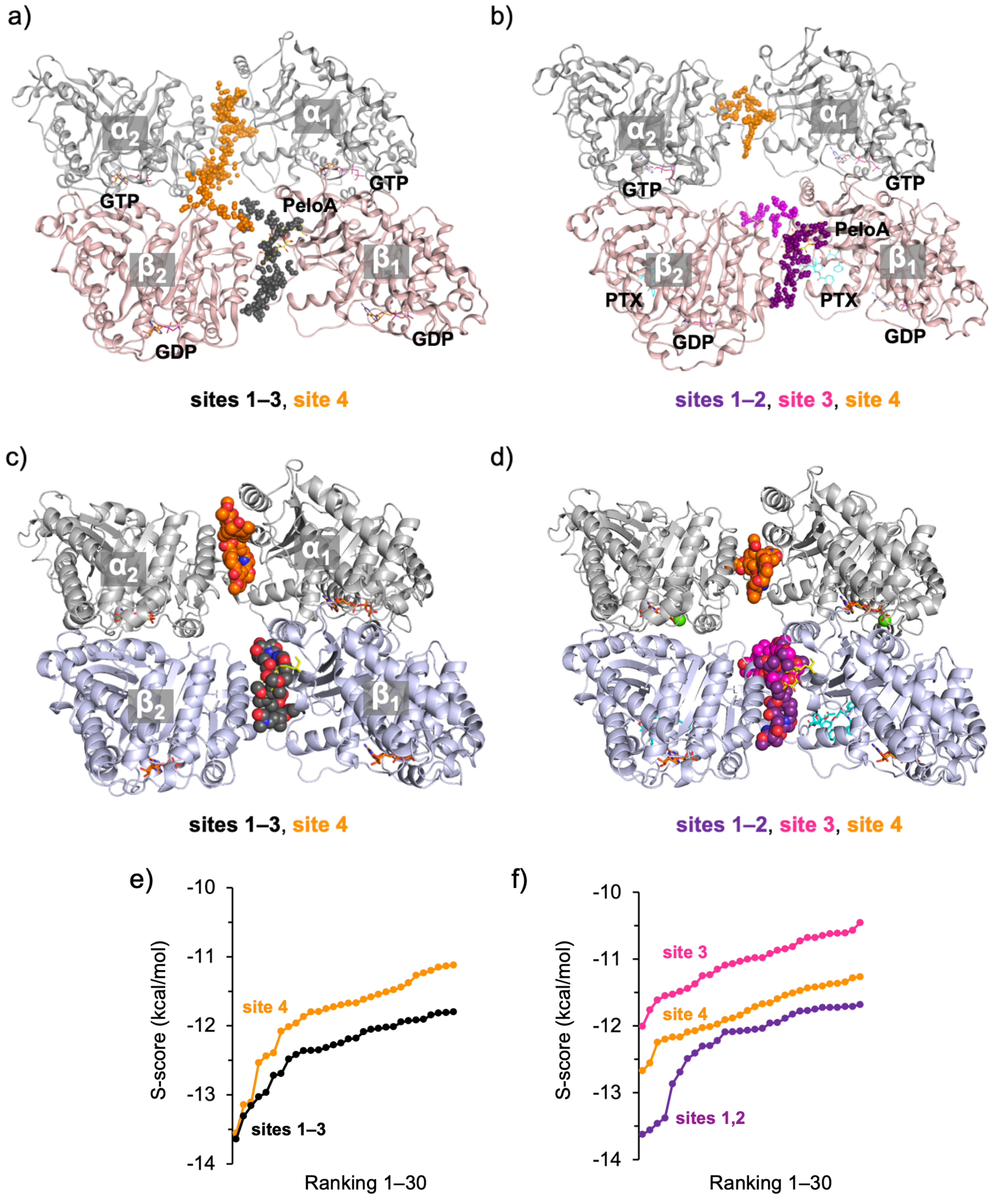

Figure S7. Docking simulation of MyC (**3**) with the tubulin heterotetramer prepared from the cryo-EM structures of the MT–peloruside A complex in the absence (PDB: 5SYC) or presence (PDB: 5SYE) of paclitaxel. a,b) Overlayed structures of the top binding sites on the tubulin heterotetramer. The ligand binding sites 1–3 (black) and 4 (orange) in a) and the sites 1–2 (purple), 3 (magenta), and 4 (orange) in b) located between the two α/β-heterodimer pairs are highlighted in sphere models, along with the binding sites for paclitaxel (teal) and GTP/GDP (red). c,d) Superposition of the lowest-energy conformers of MyC at sites 1~4 on tubulin heterotetramer. e,f) Posing and scoring of the top 30 docking models of sites 1–4 based on the S-scores.

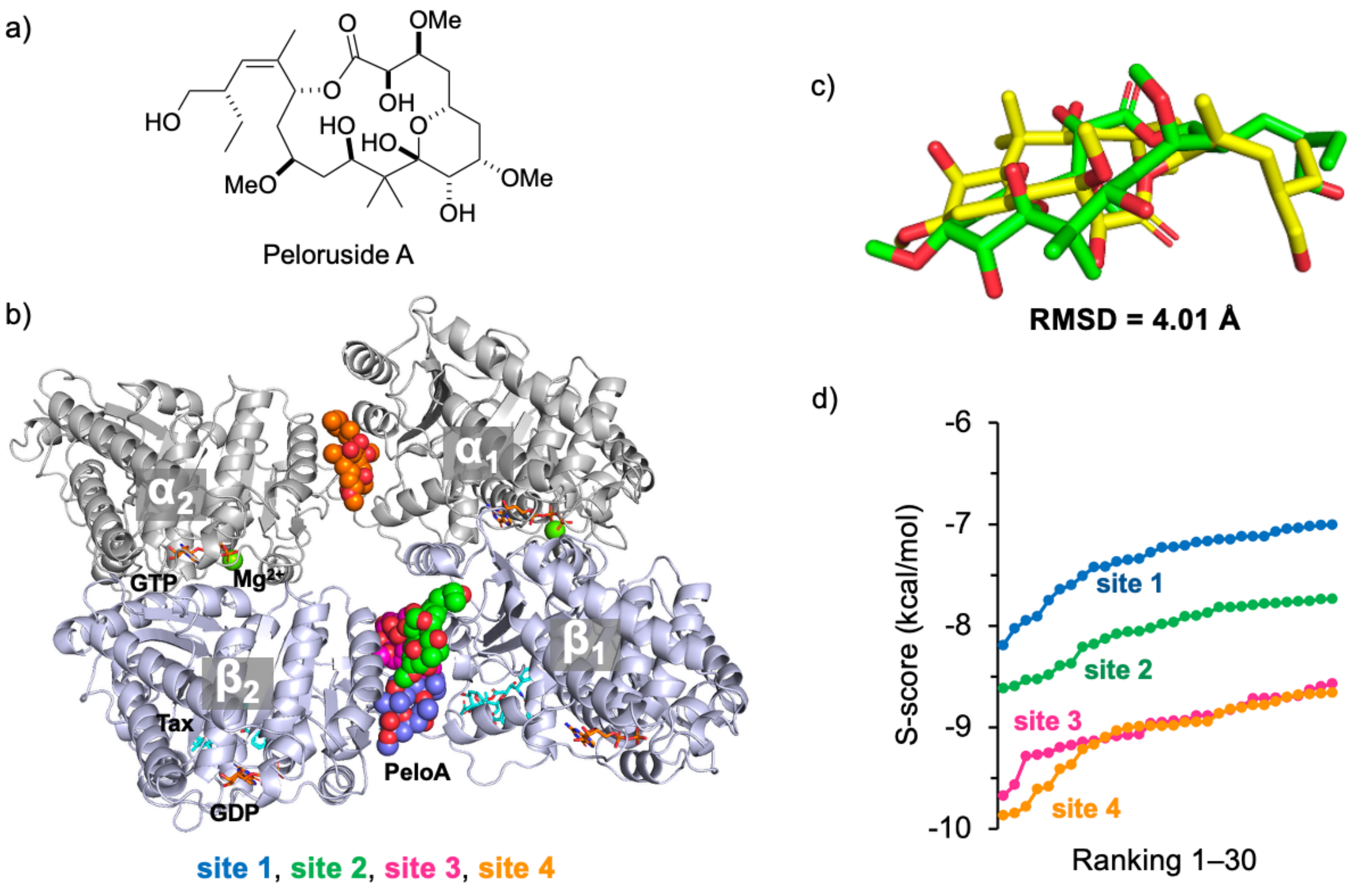

Figure S8. Docking simulation of peloruside A with the tubulin heterotetramer prepared from the cryo-EM structure of MT with paclitaxel (PDB: 5SYF). a) Structure of peloruside A. b) Superposition of the lowest-energy conformers of peloruside A at sites 1~4 on tubulin heterotetramer. c) Superposition of the peloruside A structures in the site 2 model in a) (green) and in the cryo-EM structure of MT (PDB: 5SYC) (yellow). d) Posing and scoring of the top 30 docking models of sites 1–4 based on the S-scores.

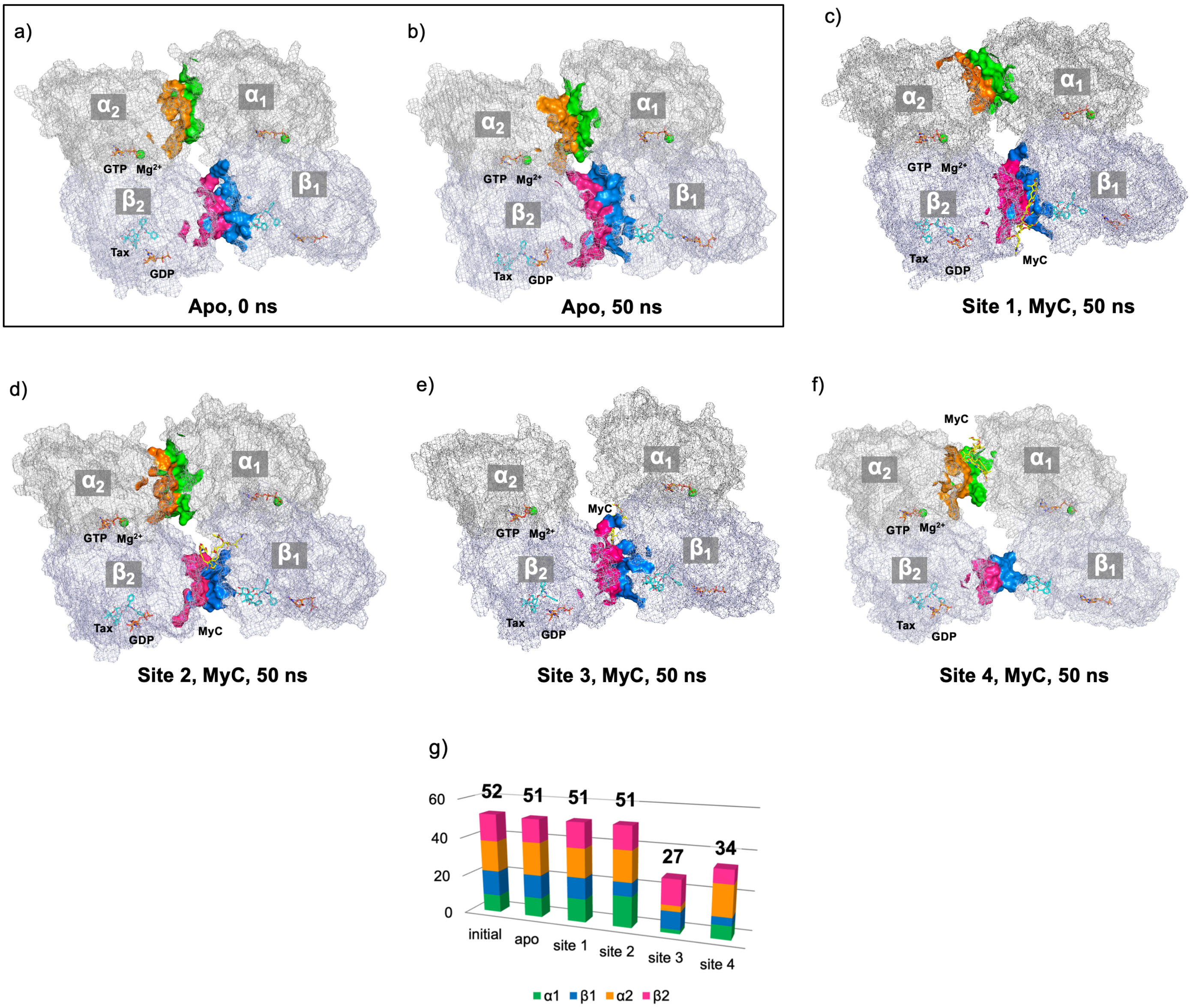

Figure S9. MD simulations of tubulin heterotetramer (PDB: 5SYF) with MyC (**3**) (yellow) for 50 ns. Paclitaxel and GTP/GDP are shown as cyan and orange sticks, respectively. a,b) Structures of tubulin heterotetramer before and after MD simulation. PPI residues between left and right tubulin α/β-heterodimers are shown as multicolor surface models. c–f) Structures of tubulin heterotetramer–MyC complex (sites 1–4) after MD simulations. g) Average numbers of the PPI residues between the left–right tubulin α/β-heterodimers on the tubulin heterotetramer (apo) and its MyC complexes (sites 1~4) over 50 ns of MD simulations.

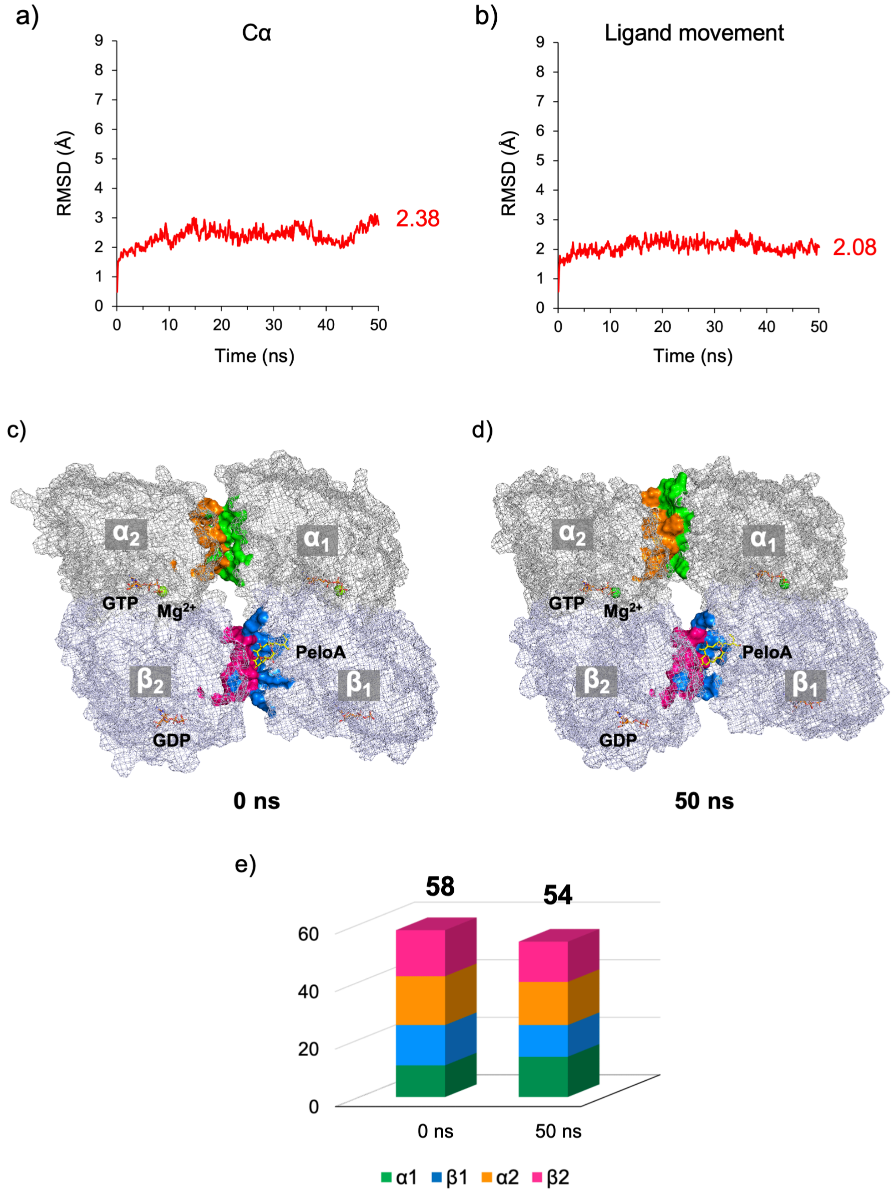

Figure S10. MD simulations of tubulin heterotetramer with peloruside A (PDB: 5SYC) for 50 ns. a) Conformational stability and b) ligand movement dynamics were simulated at 37 °C, pH 7.4 for 50 ns. c,d) Structure of the tubulin heterotetramer–peloruside A complex before and after MD simulation. Peloruside A and GTP/GDP are shown as yellow and orange sticks, respectively. PPI residues between left and right tubulin α/β-heterodimers are shown as multicolor surface models. e) Average numbers of the PPI residues between the left–right tubulin α/β-heterodimers on the tubulin heterotetramer–peloruside A complex over 50 ns of MD simulations.

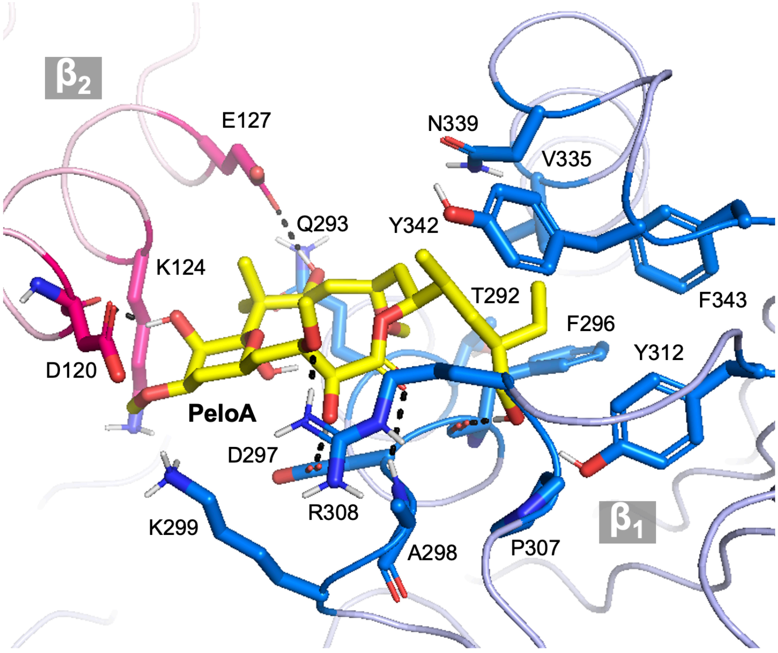

Figure S11. Detailed interactions of peloruside A (yellow) with tubulins on the cryo-EM structure of MT (PDB: 5SYC). The residues of β_1- and β_2-tubulin interact with peloruside A are shown in marine and hot pink stick models.

Table S1. Synergistic growth inhibitory effects of trisoxazole macrolactones and paclitaxel on cancer cells.

Compound	Additive	IC_{50} (nM)		
		A549	MCF-7	L1210
Paclitaxel	–	3.5	2.1	2.0
MyC (**3**)	–	25	32	11
(*E*)-**6**	–	>1000	>1000	>1000
(*Z*)-**6**	–	>1000	>1000	>1000
Paclitaxel	100 pM **3**	3.1	1.8	1.9
	100 nM (*E*)-**6**	3.5	2.4	–
	300 nM (*E*)-**6**	–	–	2.2
	100 nM (*E*)-**6**	3.5	1.8	–
	300 nM (*Z*)-**6**	–	–	3.1

Materials and Methods

General. NMR spectra were recorded on a Bruker Biospin AVANCE NEO 400 spectrometer (400 MHz for ^{1}H). For the quantification by ^{1}H NMR analysis, 4 μL of benzene solution (30 mM in CD_3OD) was added to the sample solution as a standard. Chemical shifts are reported in parts per million (ppm) relative to the solvent peak δ_H 3.31 (residual $C\underline{H}D_2OD$). Coupling constants (*J*) are shown in hertz. High-resolution electrospray ionization mass spectra (HR-ESIMS) were measured on an Agilent 6220 TOF spectrometer. Matrix-assisted laser desorption/ionization with time-of-flight mass spectrometry (MALDI-TOF MS) and tandem MALDI MS/MS analysis was performed using a Bruker UltrafleXtreme spectrometer equipped with a 355 nm Nd:YAG laser (Smartbeam 1000 MHz), with α-cyano-4-hydroxycinnamic acid (α-CHCA) as matrix, as mentioned previously.[58,63] Natural mycalolide C was kindly provided by Prof. Emer. Shigeki Matsunaga (The University of Tokyo). All chemicals were used as obtained commercially unless otherwise noted. Merck precoated silica gel $60F_{254}$ plates were used for thin layer chromatography (TLC). To protect diazirine derivatives from the light, all experiments were conducted with light-shaded glass or plastic equipment.

Cell culture and cytotoxicity. Human colon cancer HCT-116, lung cancer A549, and breast cancer MCF-7 cells were obtained from National Institutes of Biomedical Innovation, Japan (JCRB1408, JCRB0076, JCRB0134). Murine leukemia L1210 and fibroblast 3T3-L1 cells were also obtained from the same institute (JCRB9026 and JCRB9014). HCT-116, A549, and 3T3-L1 cells were maintained in Dulbecco's modified Eagle medium (DMEM) [with 4.5 g/L glucose (1.0 g/L for 3T3-L1 cells)] supplemented with 10% fetal bovine serum (FBS, Sigma–Aldrich, cat. 173012), 2 mM L-glutamine, and Antibiotic-Antimycotic Mixed Stock Solution (100 ×, 1%, Nacalai Tesque, Cat. 02892-54) in a humidified atmosphere with 5% CO_2 at 37 °C. For MCF-7 cells, the above DMEM medium was further supplemented with 1 mM sodium pyruvate and 10 μg/mL insulin. L1210 cells were maintained in Fischer's medium (with 2 mM L-glutamine) supplemented with 10% horse serum (Thermo cat. 16050130) and Antibiotic-Antimycotic Mixed Stock Solution. Cytotoxicity was measured by the 3-(4,5-dimethylthiazol-2-yl)-2,5-diphenyl tetrazolium bromide (MTT) method, as mentioned previously.[54] In brief, cells were seeded at 2×10^3 cells per well in 96-well plates. After incubation overnight at 37 °C, the cells were incubated with samples at 37 °C for 72 h (or 96 h for MCF-7 cells). A 1.4 mg/mL MTT solution in phosphate buffer saline (PBS) (50 μL) was added to the cells. After 2–4 h, the culture medium was removed, and the formazan product was dissolved in DMSO (150 μL). Optical density at 540 nm was measured with a TECAN microplate reader (Infinite® 200 Pro). For the suspended L1210 cells, cytotoxicity was measured with the WST-8 assay kit (Dojindo Laboratories, Kumamoto, Japan), and optical density at 450 nm was measured. [S1] All assays were performed in duplicate to confirm reproducibility.

Preparation of cell lysate, photolabeling, and affinity purification with photoaffinity biotin probe. HCT116 cells incubated for 4~5 days to approximately 80~90% confluence in culture flasks (1.5×10^6 cells) were washed with PBS, and treated with 0.25% trypsin–EDTA (Nacalai Tesque, cat. 35554-64). Suspended cells were collected by centrifugation and washed twice with ice-cold PBS. The cytosol fraction proteins of the cells were prepared by using EzSubcell Extract kit (ATTO Co.) with a concentration of 6~8 mg protein/mL, according to the manufacturer's instructions. The protein concentration of the cell lysate was determined by TaKaRa BCA Protein Assay Kit with BSA as a standard.

The lysate was pretreated with Neutravidin agarose (cat. 29202, Thermo Scientific) equilibrated with PBS-T in a rotary tool at 4 °C for 1 h to remove intrinsic biotin-binding proteins. The lysate (50 μL, 1.0 mg protein/mL) was incubated with MyC photoaffinity biotin probe (**5**) (1 nmol) in the presence or absence of MyC (**3**) (20 nmol) at 4 °C for 2 h. After irradiation with UV light (365 nm) with an LED365 benchtop spotlight (22 mW / cm^2 for 50 mm distance, OptoCode Co., cat. LED365-

SPT) for 1 min on ice,[60] Neutravidin agarose equilibrated with 0.1% Tween 20 in PBS (PBS-T) (10 μL) was added, and the resulting mixture was incubated with a rotator at 4 °C for 2 h. After thoroughly washed with PBS-T (4 × 100 μL) followed by PBS (100 μL), the resin was resuspended in 2 × SDS buffer (30 μL) and the photolabeled proteins were eluted by boiling at 95 °C for 5 min. SDS-PAGE was performed with a 10% precast polyacrylamide gel (ATTO), and the gels were stained with a Silver Stain Kit, Protein (GE Healthcare). Or instead, proteins in the gels after electrophoresis was transferred to PVDF membranes, using WSE-4115 Powered Blot Ace system (ATTO). Photolabeled proteins were treated with streptavidin-HRP (1:4000, Cytiva, cat. RPN1231VS), and the HRP-conjugated bands were visualized with an ImmunoStar LD kit (Wako), and detected by a Fujifilm LAS-1000 imaging scanner.

Photolabeling experiments using purified proteins. Tubulin from porcine brain (Cytoskeleton Inc., cat. T240) was reconstructed in cold RB buffer [80 mM PIPES·Na (pH 6.9), 2 mM $MgCl_2$, 0.5 mM EGTA, 1 mM GTP] at 10 mg/mL. Actin from rabbit skeletal muscle (0.20 nmol, 8.4 μg protein, Sigma Co., cat. A2522) and/or tubulin (0.20 nmol, 22 μg protein as α/β-heterodimer) prepared as above were incubated with **5** (0.20 nmol) in 300 μL of tubulin assay buffer [80 mM PIPES·Na (pH 6.9), 2 mM $MgCl_2$, 0.5 mM EGTA, 10 μM DAPI] [51] for 30 min on ice. After the irradiation with UV light (365 nm) for 1 min on ice, the resulting mixture was treated with Neutravidin agarose resin equilibrated with PBS-T (20 μL). Affinity purification of probe-bound proteins and their detection was conducted as mentioned above. For immunoblot analyses, the PVDF membrane was blocked with 0.5% skim milk in PBS-T for 1 h at room temperature. Proteins were detected with mouse monoclonal anti-α-tubulin monoclonal antibody DM1A (1:1000, Santa Cruz Biotechnology, cat. sc-32293) or rabbit polyclonal anti-β-tubulin (1:2000, Proteintech Group Inc., cat. 10094-1-AP) in 3% BSA in PBS. To detect these primary antibodies, proteins were further treated with HRP-conjugated anti-rabbit IgG (1:4000, Cytiva, cat. NA934) or anti-mouse IgG (1:2000, Cytiva, cat. NA931) in 2% skim milk in PBS.

In-del digestion and PMF analysis. [51] After SDS-PAGE, gels were silver-stained in a mass-compatible manner, according to the Bruker MALDI instrumental protocol. Stained spots were excised from the PAGE gel, the gels were desilverized and dehydrated, and the proteins were carbamidomethylated with DTT and iodoacetamide. The gels were incubated with a sequence-grade, modified trypsin (1:100 w/w, #V5111, Promega) in 25 mM ammonium bicarbonate for 17 h at 37 °C. The tryptic peptide mixtures were extracted from the gel and desalted using a ZipTip with 0.6 μL C_{18} resin (Millipore), according to the manufacturer's instruction for MS and MS/MS analyses.

In vitro F-actin and microtubule sedimentation assay by ultracentrifugation. To a solution of 6 μM tubulin in BRB80 buffer [80 mM PIPES·Na (pH 6.9), 1 mM $MgCl_2$, 1 mM EGTA] [S2,S3] (50 μL) were added paclitaxel (0.6 nmol) to induce MT formation, and/or 6 μM actin in BRB80 buffer (50 μL) and MyC (**3**) (3 nmol). After incubation for 30 min at 37 °C, samples were ultracentrifuged (150,000 × *g*, 25 °C, 1 h).[51,64] Aliquots of the supernatants and precipitates (redissolved in BRB80 buffer) were mixed with the same amount of 2× SDS buffer and boiled for 5 min at 95 °C. SDS-PAGE was performed using a precast 10% polyacrylamide gel, and the gels were stained with a Quick-CBB kit (Wako).

Fluorescence-based tubulin polymerization assay. [51,65] To the ×10 samples (5 μL, including paclitaxel, mycalolide analogs, and actin) in a half-volume 96-well plate were added 50 μL aliquots of 2 mg/mL tubulin (20 μM for the heterodimer) in assay buffer [80 mM PIPES·Na (pH 6.9), 2 mM $MgCl_2$, 0.5 mM EGTA, 1 mM GTP, 4.6 μM DAPI] cooled on ice, according to the manufacturer's instruction for this assay kit (cat. BK011P, Cytoskeleton Inc.). Tubulin polymerization was monitored by the increase in fluorescence ($\lambda_{ex/em}$ 360/465 nm) at 37 °C, using a TECAN Infinite 200®$_{PRO}$ fluorescent microplate reader. The

fluorescence intensity at which MTs were nearly completely polymerized by adding 20% glycerol (v/v) to the assay buffer was taken as 100%, and the relative tubulin assembly of each sample was calculated.

Negative stain and TEM imaging analysis. To a solution of 3 μM tubulin (0.33 mg/mL) in modified RB buffer [80 mM PIPES·Na (pH 6.9), 1 mM $MgCl_2$, 1 mM EGTA] (50 μL) were added paclitaxel (0.3 nmol) and/or mycalolide analogs (0.43 nmol). After incubation for 30 min at 37 °C, the MTs were negatively stained with 2% uranyl acetate and observed with a JEM-1010 electron microscope (JEOL) at an accelerating voltage of 100 kV, similarly as described previously.[S4] Diameter of MT was measured by using ImageJ software, and the data are expressed as the mean ± SD (n=10).

Molecular modeling studies. Molecular modeling studies were performed using the Molecular Operating Environment (MOE) 2024.06 program package (Chemical Computing Group, Inc.), similarly as described previously.[53,S5] For docking model studies, all water molecules associated with the cryo-EM reconstructions of paclitaxel-stabilized (PDB: 5SYF),[69] peloruside A-stabilized (PDB: 5SYC),[69] or paclitaxel/peloruside A-stabilized (PDB: 5SYE) [69] porcine MT complexes were removed, except for those near the ligand, and all protons on the protein and the ligand were complemented. On the docking simulations of MyC or peloruside A with the two pairs of α/β-tubulin heteodimer (tubulin heterotetramer), ten ligand pockets were generated by the Site-Finder mode, and four of them, excluding six in which paclitaxel and GTP/GDP bind, were settled as the ligand-binding sites 1–4. Conformational searches were performed using the Amber14:EHT force-field with GB/VI Generalized Born implicit solvent electrostatics (D_{in} = 1, D_{out} = 80) and with LowModeMD. Refinements were performed using the induced-fit model, and the lowest conformation models in energy in each site were used for further MD simulation studies. The top 300 minimized binding poses obtained were prioritized based on the S values, which indicate the binding stability between the target proteins and the ligands.

Molecular dynamics simulation. Molecular dynamics (MD) simulations were performed for the tubulin heterotetramer–MyC (or peloruside A) complexes and its apo form using YASARA (21.12.19) software to evaluate the conformational stability of proteins and ligands.[53,S5,S6] The complexes were inserted into a cubic box of water molecules, with a density of 0.997 g/mL and a temperature of 310 K to mimic the cellular environment. We used 0.9% NaCl (physiological solution) and a default physiological pH at 7.4. The simulation was performed at a normal speed (2 × 1.25 fs timestep). After the steepest descent and simulated annealing minimizations, a simulation was run for 50 ns using the AMBER14 Force Field. The water molecules were described by the TIP3P model. All protein structural data were represented in cartoon and surface model, while ligand was showed as sphere or stick model. MOE and PyMol (Molecular Graphics System, Version 2.0 Schrödinger, LLC) were used for final image processing which suitable for scientific representation.

Synthesis and spectroscopic data of MyC photoaffinity probe.

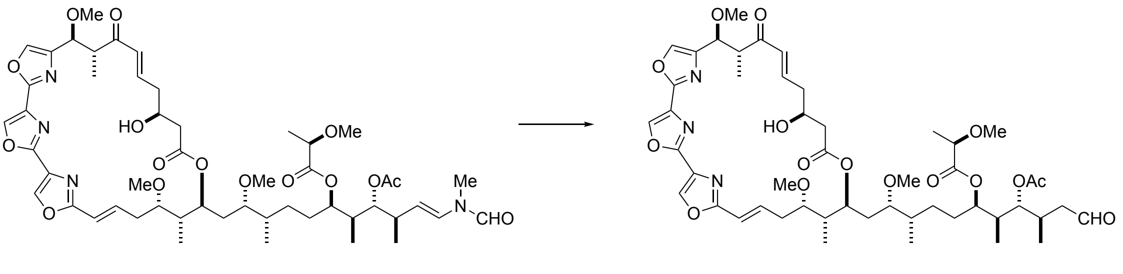

MyC (**3**) MyC aldehyde (**3a**)

MyC aldehyde (**3a**). A solution of MyC (**3**, 0.6 mg, 600 nmol) in a 1:1 mixture of MeCN–1 M HCl aq. (0.4 mL) was stirred for 1 h at 60°C.[20,30] The resulting mixture was diluted with sat $NaHCO_3$ aq. (2 mL) and brine (2 mL), and extracted with EtOAc (2 mL × 3). The combined extracts were dried with anhydrous Na_2SO_4 and concentrated to give MyC aldehyde (**3a**), which was used for next step without further purification. **3a**: R_f = 0.34 ($CHCl_3$/MeOH = 9/1); HRMS (ESI) *m/z* 978.4585 (calcd for $C_{49}H_{69}N_3NaO_{16}$ $[M+Na]^+$, Δ +1.4 mmu).

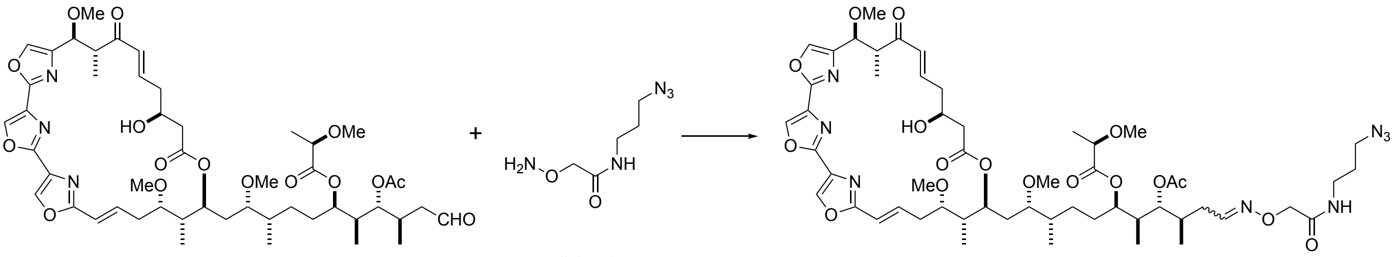

MyC aldehyde (**3a**) linker **8** MyC azide (**3b**)

MyC azide (**3b**). MyC aldehyde (**3a**) prepared as above was dissolved in 2 mM solution of alkoxyamine **8** [S7] in EtOH (0.8 mL, 1.6 μmol), and PPTS (3.0 mg, 12 μmol) was added. After stirring for 24 h at room temperature, the resulting mixture was quenched with acetone (50 μL). After 30 min, the reaction mixture was concentrated and directly applied to a Develosil ODS-HG-5 HPLC column (ϕ 20 mm I.D. × 250 mm). Samples were eluted with 75% MeOH aq. at a flow rate of 5 mL/min, with monitoring at 254 nm to give MyC azide (**3b**) (98 nmol, 16% in 2 steps based on NMR quantification, *E*/*Z* = 2/1 for the C35 isomers). **3b**: t_R = 19.9, 20.8 min [Develosil ODS-HG-5 (ϕ 4.6 mm × 250 mm), 60% aq. MeCN isocratic for 30 min, 1.0 mL/min, 25°C, UV: 254 nm]; ^{1}H NMR (400 MHz, CD_3OD) δ 8.57 (s, 1H), 8.49 (s, 1H), 8.04 (s, 1H), 7.58 [6.87] (t, *J* = 5.9 [5.3] Hz, 1H), 7.40 (m, 1H), 7.09 (m, 1H), 6.47 (d, *J* = 15.4 Hz, 1H) , 6.17 (d, *J* = 15.6 Hz, 1H), 5.30 (t, *J* = 7.8 Hz, 1H), 5.06 (t, *J* = 7.2 Hz, 1H), 4.76 (m, 1H), 4.47 (s, 1H), 4.42 (s, 2H), 4.35 (d, *J* = 9.4 Hz, 1H), 4.24 (m, 1H) , 3.85 (q, *J* = 6.9 Hz, 1H), 3.62 (m, 1H), 3.52 (s, 6H). 3.37–3.33 (m, 5H), 3.30–3.20 (m, 2H), 3.17 (s, 3H), 3.12 (m, 1H), 2.79–2.46 (m, 8H), 2.041 [2.047] (s, 3H), 1.89–1.75 (m, 2H), 1.75 (m, 2H), 1.65–1.29 (m, 5H), 1.17 (m, 2H), 1.02 [1.00] (d, *J* = 5.5 [5.5] Hz, 3H), 0.987 [0.982] (d, *J* = 8.9 [8.4] Hz, 3H), 0.932 (d, *J* = 6.7 Hz, 6H), 0.88 [0.87] (d, *J* = 6.8 [6.8] Hz, 3H); Chemical shifts of the minor *Z*-isomer are within parentheses (square brackets); HRMS (ESI) *m/z* 1133.5379 (calcd for $C_{54}H_{78}N_8NaO_{17}$ $[M+Na]^+$, Δ +0.1 mmu).

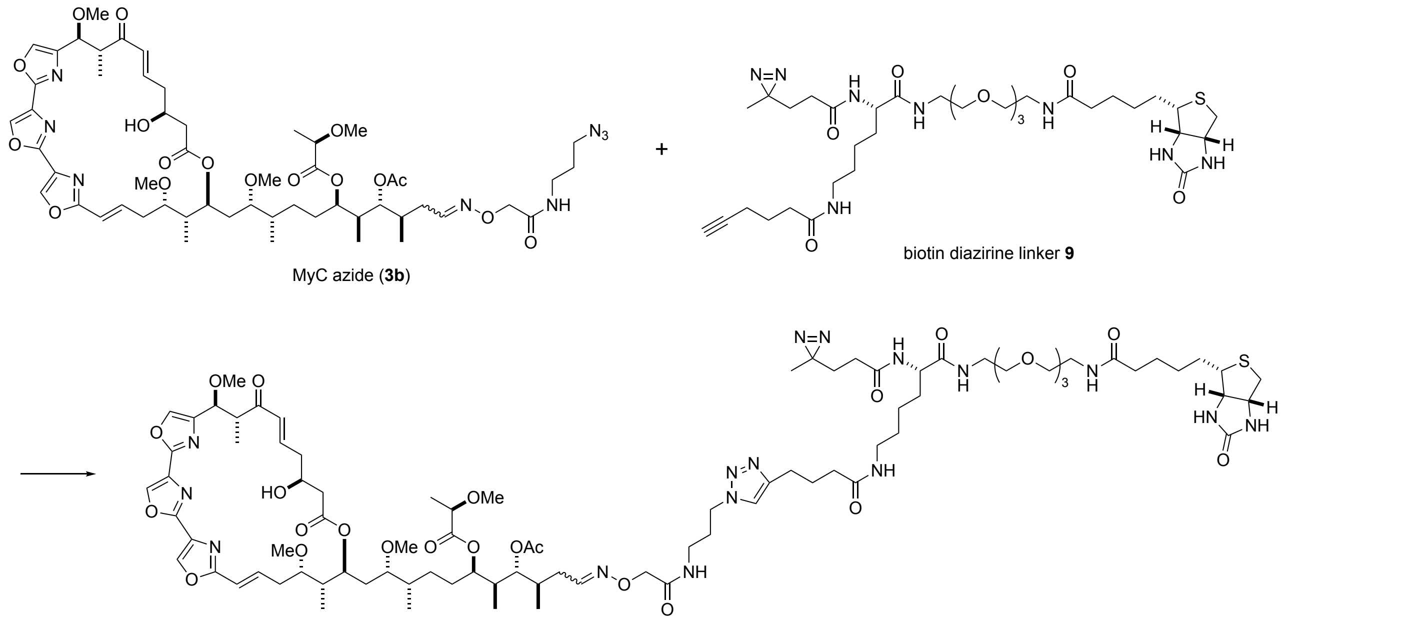

MyC photoaffinity biotin probe (5)

MyC photoaffinity biotin probe (5). To a stirred solution of 4.7 mM biotin diazirine linker **9** [60] (8 μL, 25 nmol) in ^{t}BuOH and MyC azide (**3b**) (20 nmol) were added 40 mM $Cu(OAc)_2$ aq. (2 μL, 80 nmol) and 164 mM sodium ascorbate aq. (2 μL, 330 nmol). After being stirred for 24 h at 37 °C, MeOH (1 mL) was added and filtered through a small plug of cotton. After the filtrate was concentrated, the crude material was purified with a reverse-phase HPLC [Develosil ODS-HG-5, ϕ 4.6 × 250 mm, 20–100% MeCN aq., 1 mL/min, 215 nm] to give MyC photoaffinity biotin probe (**5**) (quant. based on HPLC monitoring analysis) as a colorless oil. **5**: t_R = 6.4 min [Develosil XG-C18LC-5 (ϕ 2.0 mm × 150 mm), 85–100% aq. MeOH gradient for 30 min, 0.2 mL/min, rt]; HRMS (ESI) *m/z* 953.4695 (calcd for $C_{89}H_{136}N_{16}Na_2O_{25}S$ $[M+Na]^{2+}$, Δ +1.1 mmu).

Trisoxazole analog **7**. Prepared according to the scheme shown below, which was outlined in reference [67].

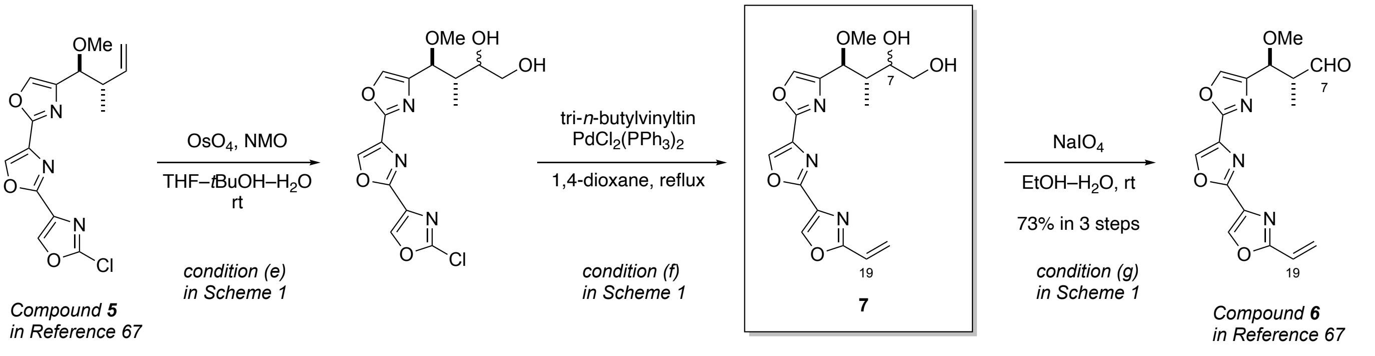

PMF analysis.

48 kDa protein

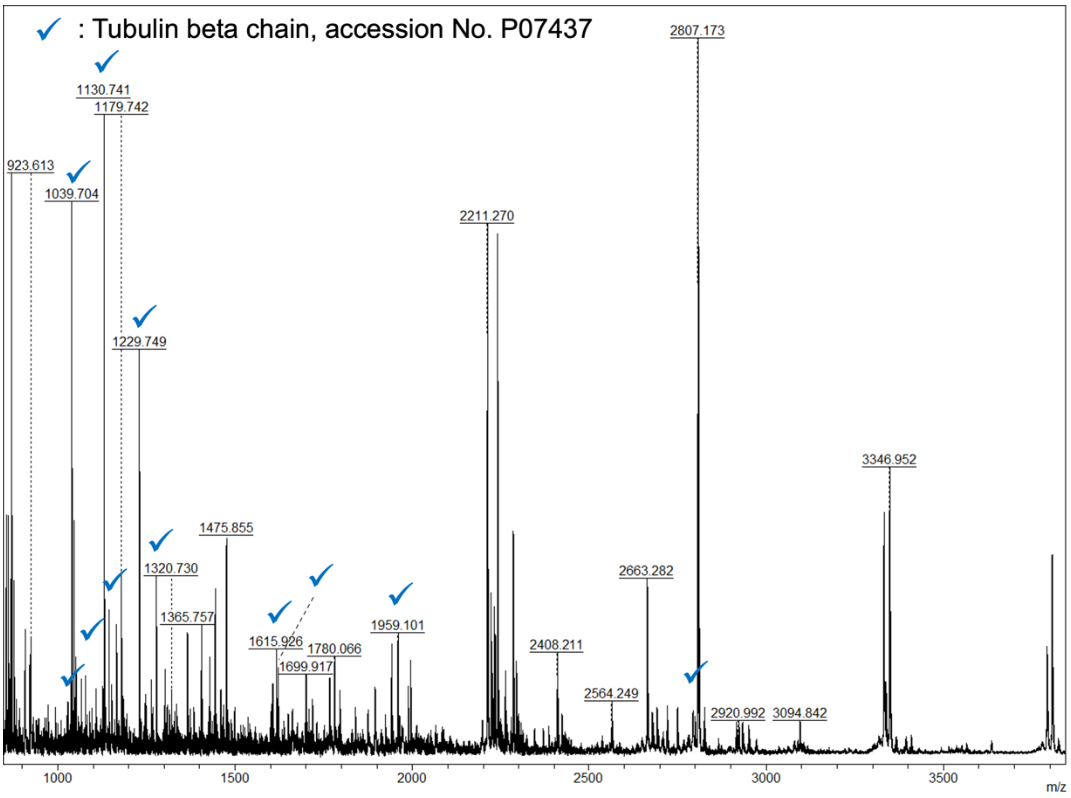

Assigned tryptic peptides

	Observed (*m/z*) [a]	Calcd (*m/z*)	Start	End	Sequence
K1	1028.61	1028.52[b]	351	359	TAVCDIPPR
K2	1039.70	1039.59	310	318	YLTVAAVFR
K3	1077.66	1077.53	155	162	IREEYPDR
K4	1130.74	1130.60	242	251	FPGQLNADLR
K5	1143.77	1143.63	253	262	LAVNMVPFPR
K6	1229.75	1229.60	381	390	ISEQFTAMFR
K7	1301.76	1301.64	47	58	ISVYYNEATGGK
K8	1615.93	1615.84	63	77	AILVDLEPGTMDSVR
K9	1620.91	1620.84	263	276	LHFFMPGFAPLTSR
K10	1959.10	1958.98	104	121	GHYTEGAELVDSVLDVVR
K11	2798.34	2800.08	78	103	SGPFGQIFRPDNFVFGQSGAGNNWAK

[a] The data represent the monoisotopic ion peak $(M+H)^+$ values.

[b] Putative values indicating that the Cys residue(s) in the sequences were carbamidomethylated.

```
        10         20         30         40         50         60         70         80         90        100        110
MREIVHIQAG QCGNQIGAKF WEVISDEHGI DPTGTYHGDS DLQLDRISVY YNEATGGKYV PRAILVDLEP GTMDSVRSGP FGQIFRPDNF VFGQSGAGNN WAKGHYTEGA

       120        130        140        150        160        170        180        190        200        210        220
ELVDSVLDVV RKEAESCDCL QGFQLTHSLG GGTGSGMGTL LISKIREEYP DRIMNTFSVV PSPKVSDTVV EPYNATLSVH QLVENTDETY CIDNEALYDI CFRTLKLTTP

       230        240        250        260        270        280        290        300        310        320        330
TYGDLNHLVS ATMSGVTTCL RFPGQLNADL RKLAVNMVPF PRLHFFMPGF APLTSRGSQQ YRALTVPELT QQVFDAKNMM AACDPRHGRY LTVAAVFRGR MSMKEVDEQM

       340        350        360        370        380        390        400        410        420        430        440
LNVQNKNSSY FVEWIPNNVK TAVCDIPPRG LKMAVTFIGN STAIQELFKR ISEQFTAMFR RKAFLHWYTG EGMDEMEFTE AESNMNDLVS EYQQYQDATA EEEEDFGEEA

EEEA
```

44 kDa protein

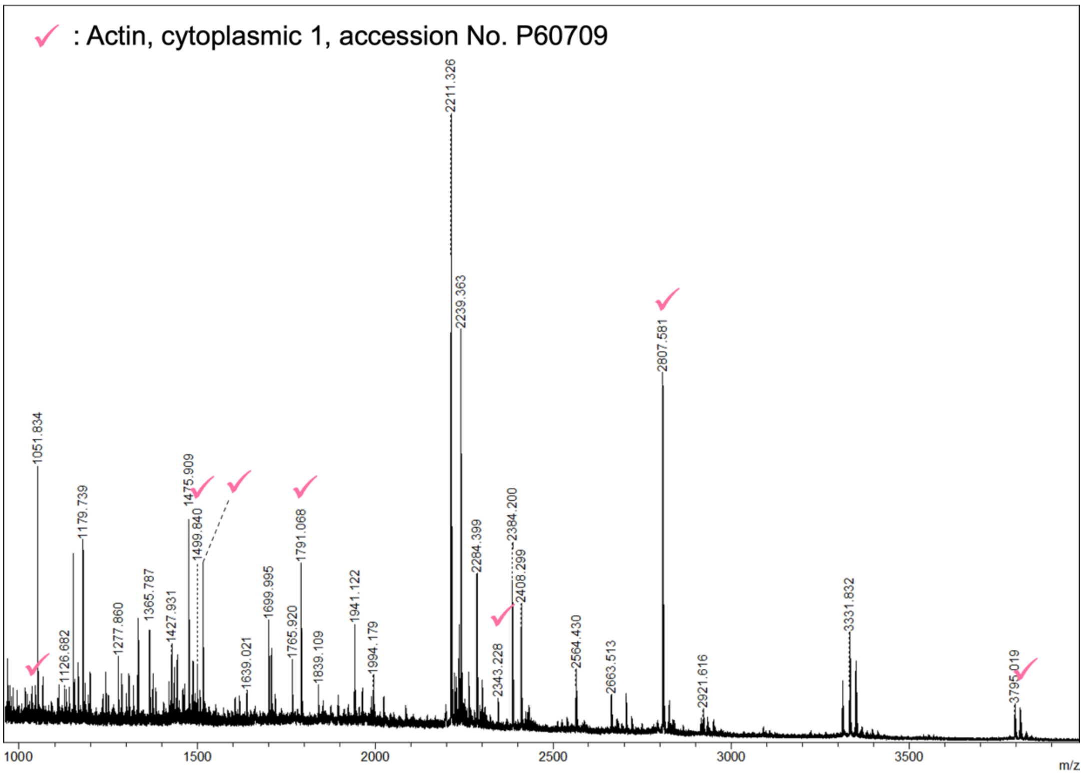

Assigned tryptic peptides

	Observed (*m/z*) [a]	Calcd (*m/z*)	Start	End	Sequence
K1	1036.65	1036.65	327	335	IKIIAPPER
K2	1499.84	1499.68 [b]	360	372	QEYDESGPSIVHR
K3	1515.92	1515.75	85	95	IWHHTFYNELR
K4	1791.07	1790.89	239	254	SYELPDGQVITIGNER
K5	2343.23	2343.16	291	312	KDLYANTVLSGGTTMYPGIADR
K6	2807.58	2807.31[c]	214	238	EKLCYVALDFEQEMATAASSSSLEK
K7	3810.04	3808.97	148	183	ITGIVMDSGDGVTHTVPIYEGYALPHAILRLDLAGR

[a] The data represent the monoisotopic ion peak $(M+H)^+$ values.
[b] *N*-terminus glutamine was converted into pyroglutamic acid.
[c] Putative values indicating that the Cys residue(s) in the sequences were carbamidomethylated.

42 kDa protein

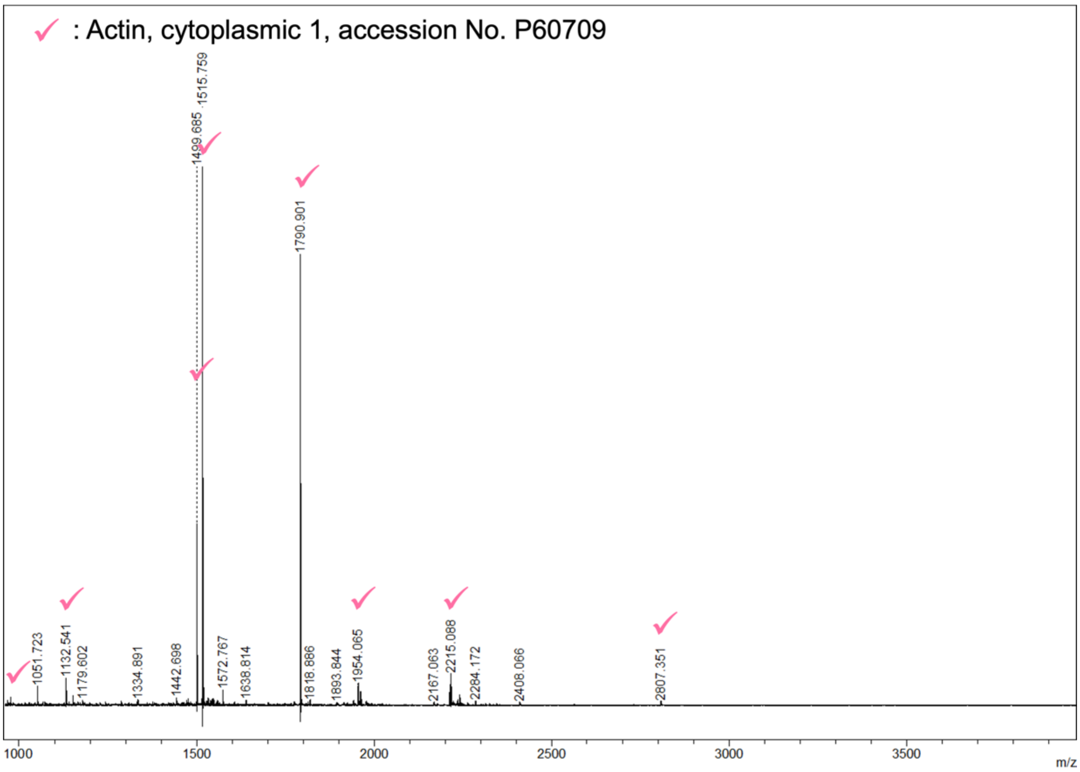

Assigned tryptic peptides

	Observed (*m/z*) [a]	Calcd (*m/z*)	Start	End	Sequence
K1	976.47	976.45	19	28	AGFAGDDAPR
K2	1132.45	1132.53	197	206	GYSFTTTAER
K3	1499.69	1499.68 [b]	360	372	QEYDESGPSIVHR
K4	1515.76	1515.75	85	95	IWHHTFYNELR
K5	1790.90	1790.89	239	254	SYELPDGQVITIGNER
K6	1954.06	1954.06	96	113	VAPEEHPVLLTEAPLNPK
K7	2215.09	2215.07	292	312	DLYANTVLSGGTTMYPGIADR
K8	2807.35	2807.31 [c]	214	238	EKLCYVALDFEQEMATAASSSSLEK

[a] The data represent the monoisotopic ion peak $(M+H)^+$ values.
[b] *N*-terminus glutamine was converted into pyroglutamic acid.
[c] Putative values indicating that the Cys residue(s) in the sequences were carbamidomethylated.

30 kDa protein

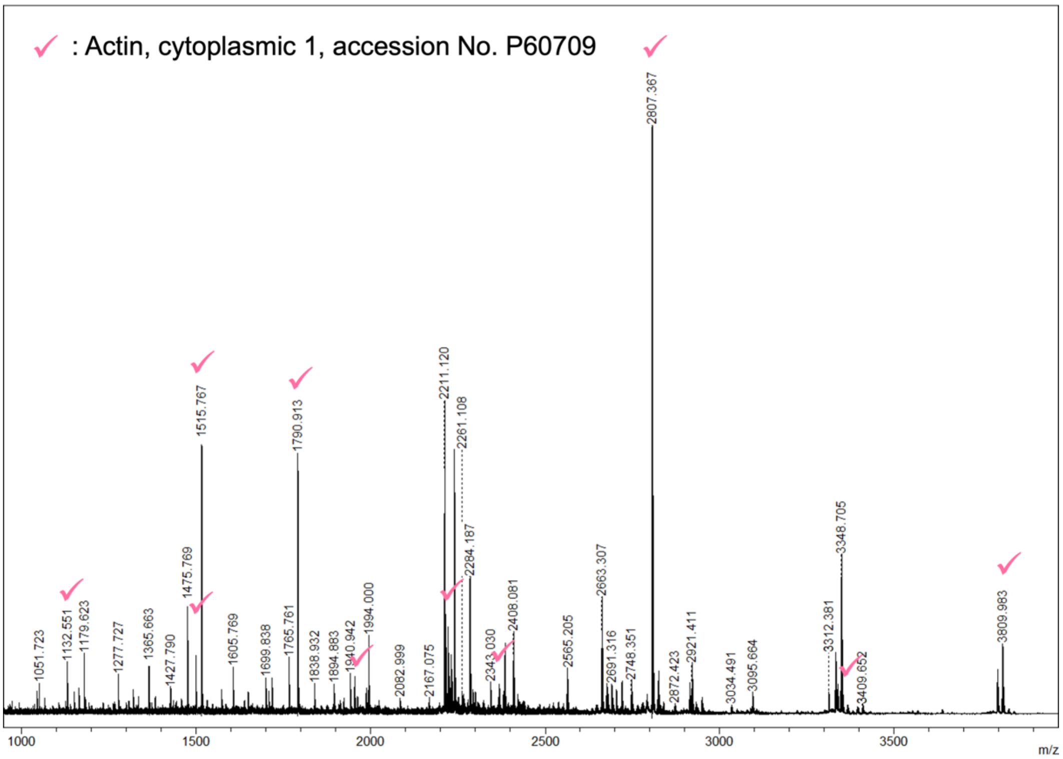

Assigned tryptic peptides

	Observed (*m/z*) [a]	Calcd (*m/z*)	Start	End	Sequence
K1	1132.55	1133.21	197	206	GYSFTTTAER
K2	1499.70	1499.68 [b]	360	372	QEYDESGPSIVHR
K3	1515.77	1515.75	85	95	IWHHTFYNELR
K4	1790.91	1790.89	239	254	SYELPDGQVITIGNER
K5	1954.07	1954.06	96	113	VAPEEHPVLLTEAPLNPK
K6	2215.08	2215.07	292	312	DLYANTVLSGGTTMYPGIADR
K7	2343.03	2343.16	291	312	KDLYANTVLSGGTTMYPGIADR
K8	2367.23	2368.85 [c]	29	50	AVFPSIVGRPRHQGVmVGMGQK
K9	2807.37	2807.31 [d]	214	238	EKLCYVALDFEQEMATAASSSSLEK
K10	3366.67	3367.83 [c]	178	206	LDLAGRDLTDYLmKILTERGYSFTTTAER
K11	3809.98	3810.31 [c, d]	277	309	GSQQYRALTVPELTQQVFDAKNmmAACDPRHGR

[a] The data represent the monoisotopic ion peak $(M+H)^+$ values.
[b] *N*-terminus glutamine was converted into pyroglutamic acid.
[c] "m" means oxidized methionine residue.
[d] Putative values indicating that the Cys residue(s) in the sequences were carbamidomethylated.

NMR spectra

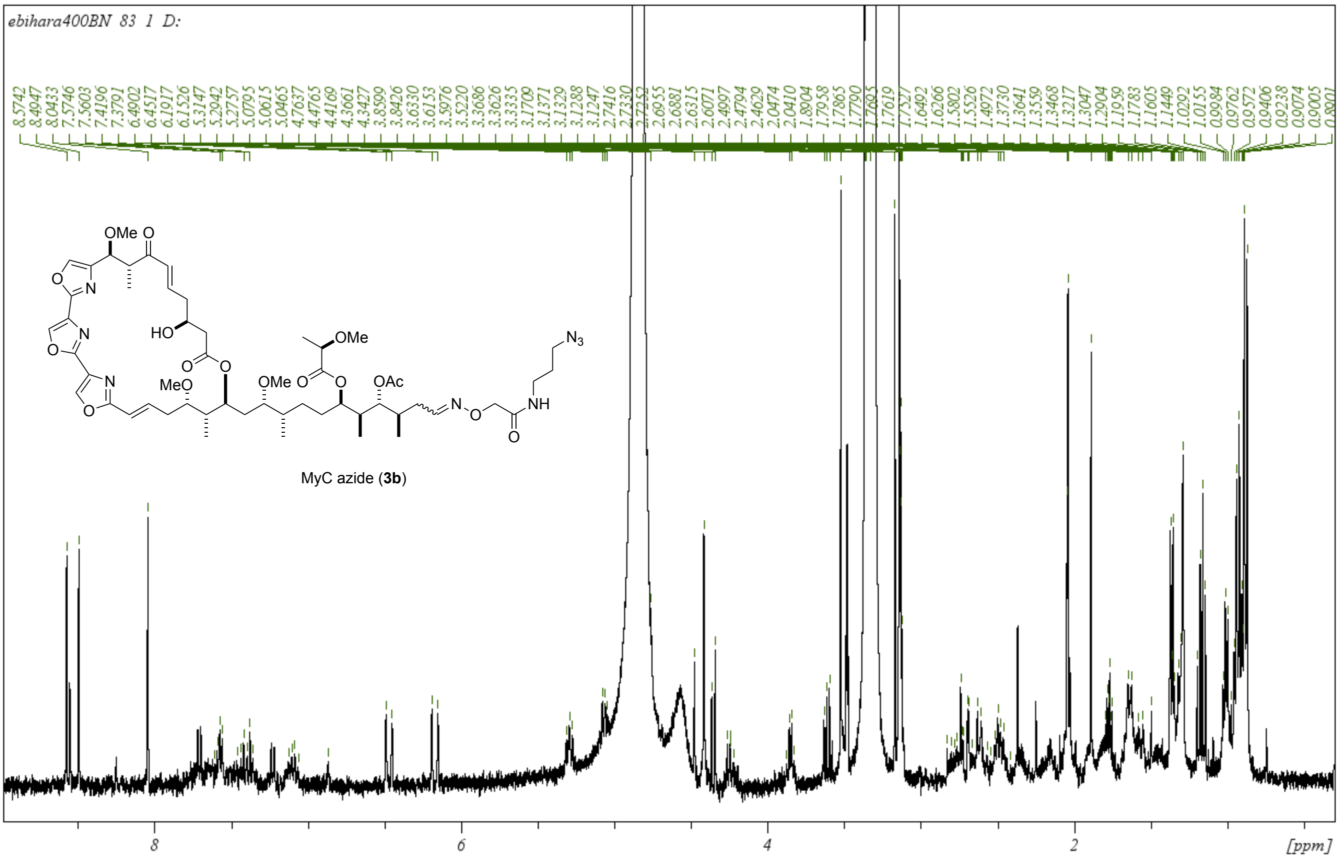

^{1}H NMR spectrum of MyC azide (**3b**) (400 MHz, CD_3OD).

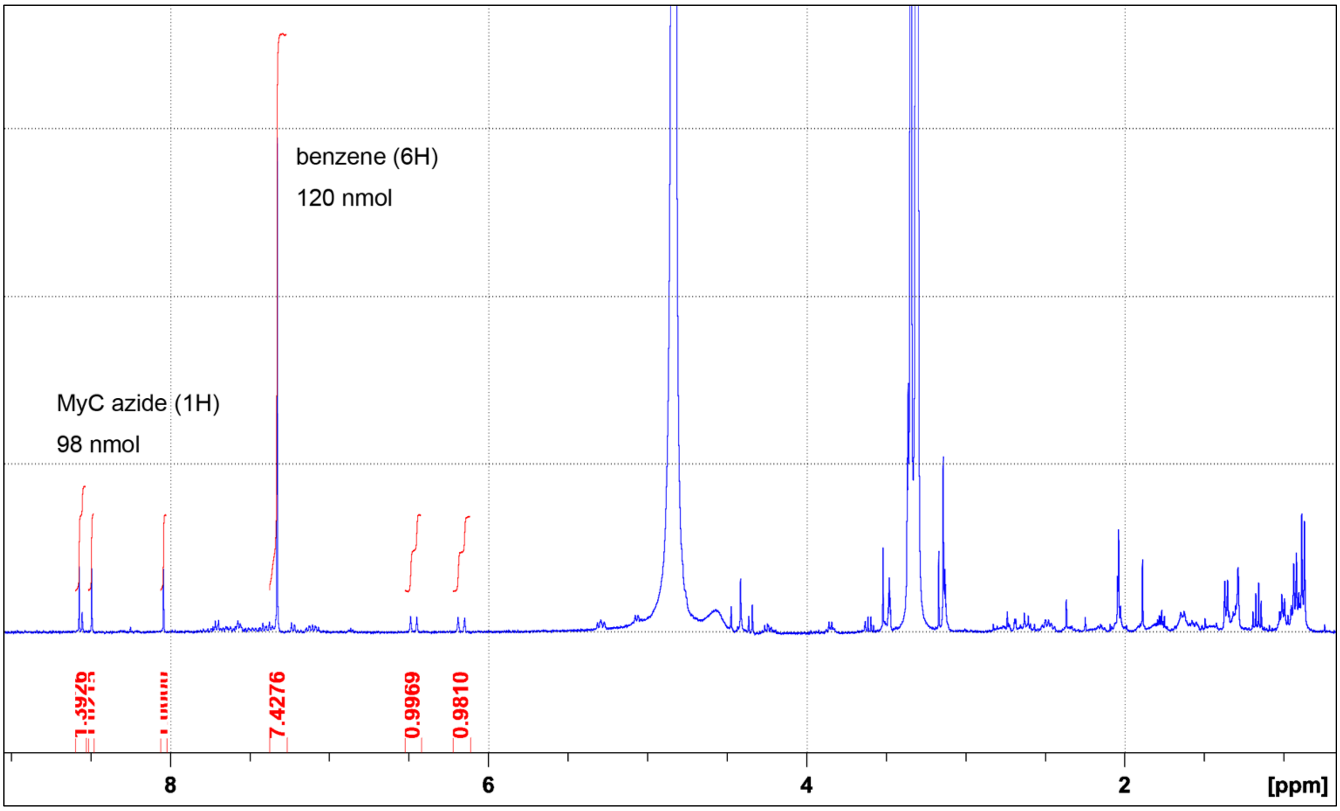

^{1}H NMR spectrum of MyC azide (**3b**) (98 nmol) with benzene (120 nmol) (400 MHz, CD_3OD).

HRMS spectra

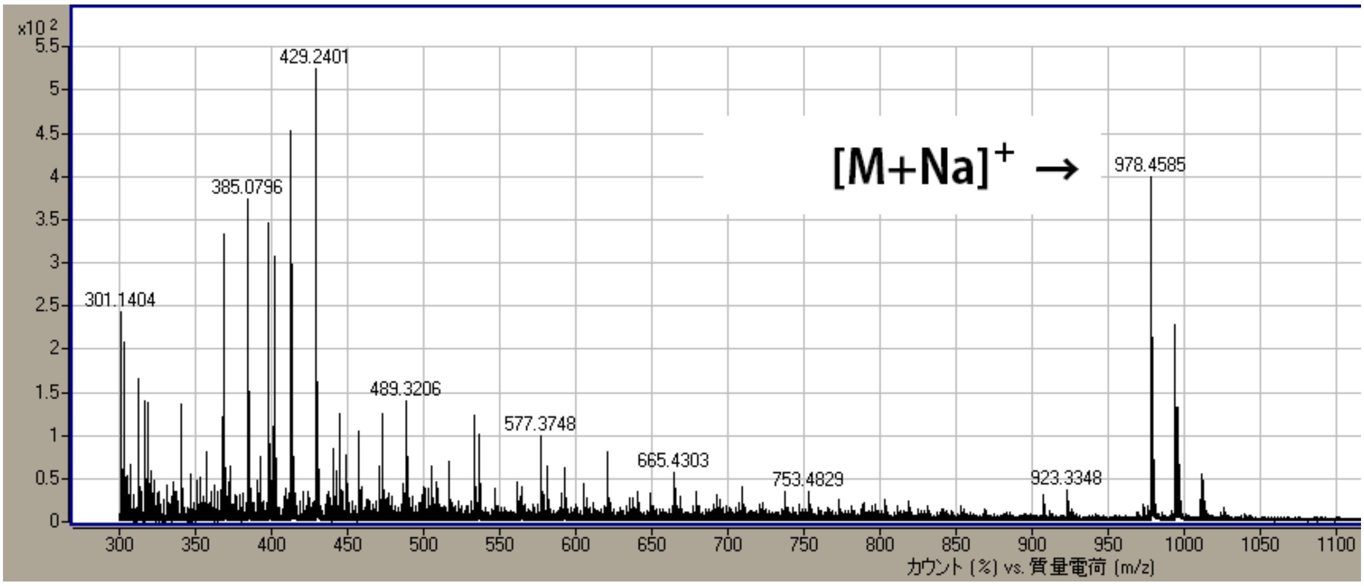

ESI mass spectrum of MyC aldehyde (**3a**).

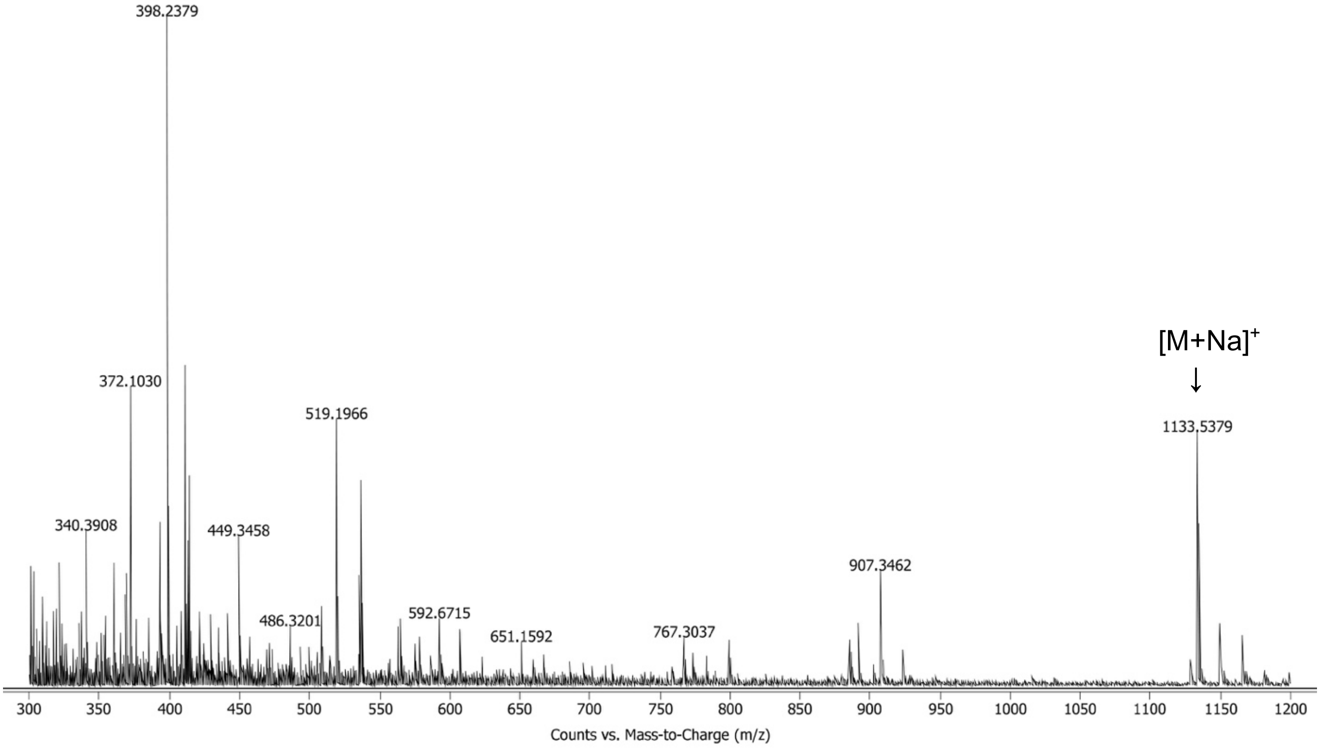

ESI mass spectrum of MyC azide (**3b**).

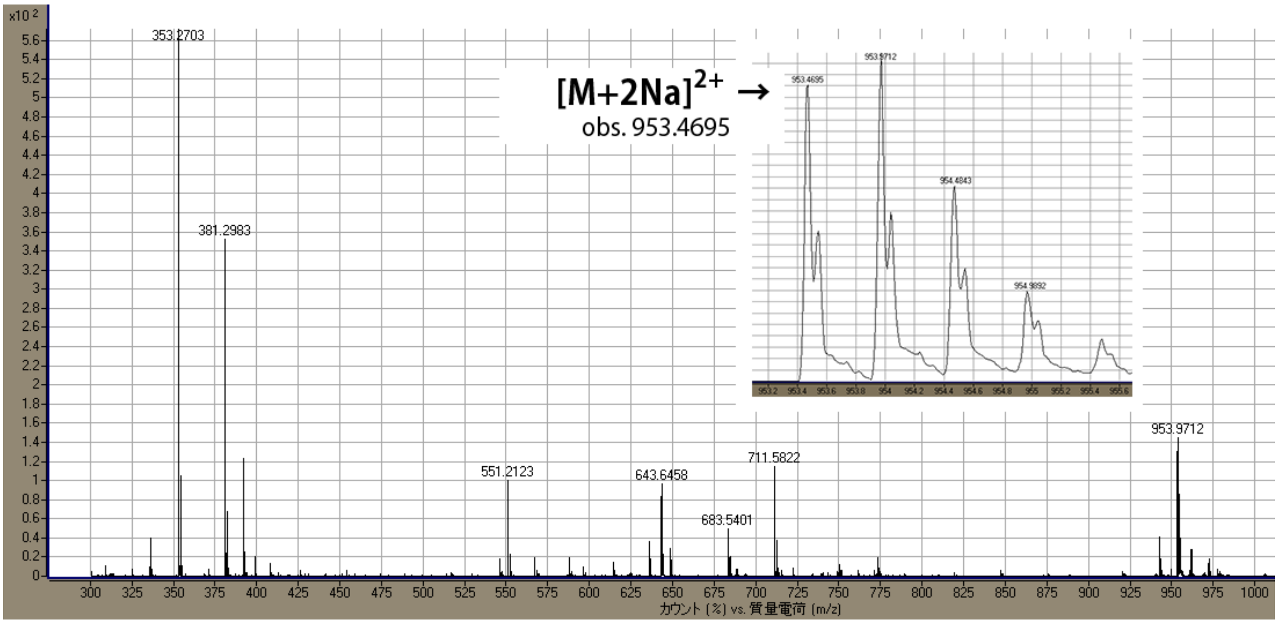

ESI mass spectrum of MyC photoaffinity biotin probe (**5**).

HPLC charts

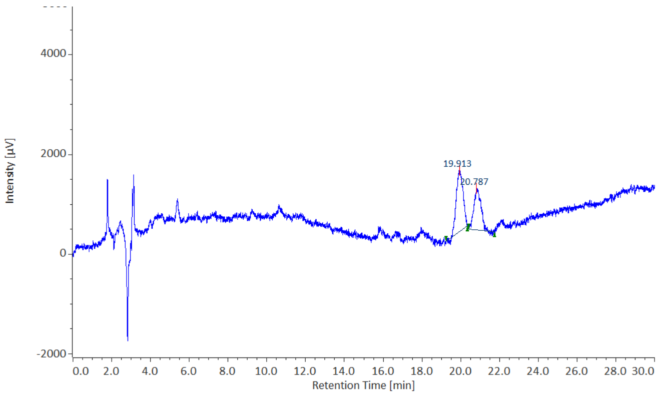

MyC azide (**3b**)

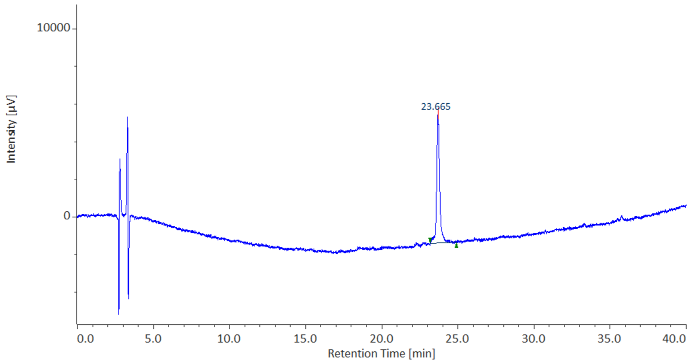

MyC photoaffinity biotin probe **5**

HPLC conditions: Develosil ODS-HG-5 (Φ 4.6 mm × 250 mm), 60% aq. MeCN isocratic for 30 min (for **3b**) / 20–100% aq. MeCN linear gradient for 40 min (for **5**), 1.0 mL/min, 25°C, UV: 254 nm.

Original gel images

Fig. 2b

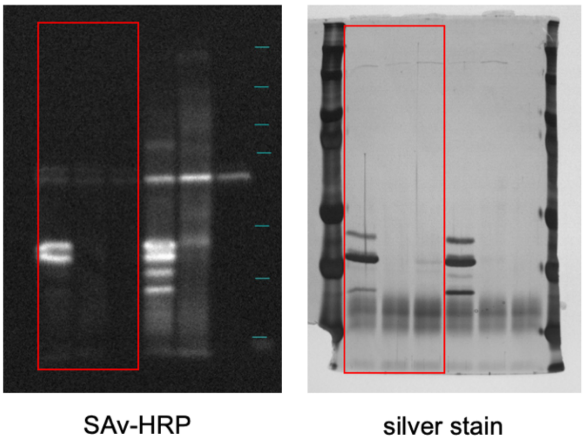

Fig. 2c

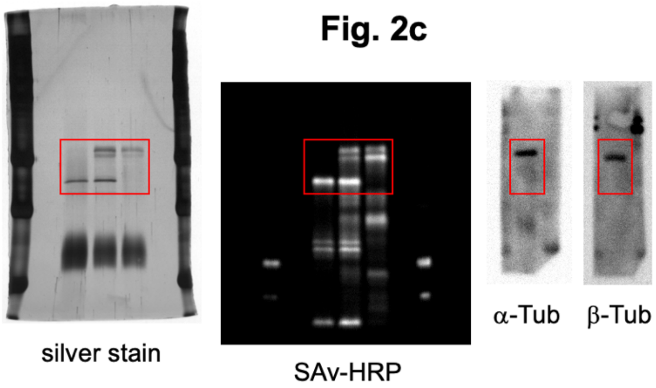

Fig. 2d

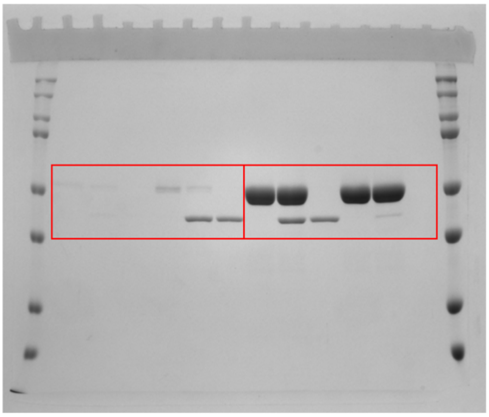

Supporting References

S1. Y. Araki, Y. Hanaki, M. Kita, K. Hayakawa, K. Irie, Y. Nokura, A. Nakazaki, T. Nishikawa, *Biosci. Biotech. Biochem.* **2021**, *85*, 1371–1382.

S2. M. L. Shelanski, F. Gaskin, C. R. Cantor, *Proc. Natl. Acad. Sci. U.S.A.* **1973**, *70*, 765–768.

S3. J. C. Lee, S. N. Timasheff, *Biochemistry* **1977**, *16*, 1754–1762.

S4. A. Oshima, T. Matsuzawa, K. Nishikawa, Y. Fujiyoshi, *J. Biol Chem.* **2013**, 288, 10513–10521.

S5. Y. Sun, A. Dakiiwa, M. Zhang, T. Shibata, M. Kita, *Chem. Eur. J.* **2024**, *30*, e202402049.

S6. R. Fukuoka, Y. Yano, N. Hara, C. Sadamoto, A. D. Maturana, M. Kita, *Angew. Chem. Int. Ed.* **2025**, *64*, e202503891.

S7. F. Tang, Y. Yang, Y. Tang, S. Tang, L. Yang, B. Sun, B. Jiang, J. Dong, H. Liu, M. Huang, M.-Y. Geng, W. Huang, *Org. Biomol. Chem.* **2016**, *14*, 9501–9518.
